# Multiple Patterns of Regulation and Overexpression of a Ribonuclease-Like Pathogenesis-Related Protein Gene, *OsPR10a*, Conferring Disease Resistance in Rice and *Arabidopsis*

**DOI:** 10.1371/journal.pone.0156414

**Published:** 2016-06-03

**Authors:** Li-Fen Huang, Kuan-Hung Lin, Siou-Luan He, Jyh-Lang Chen, Jian-Zhi Jiang, Bo-Hong Chen, Yi-Syuan Hou, Ruey-Shyang Chen, Chwan-Yang Hong, Shin-Lon Ho

**Affiliations:** 1 Graduate School of Biotechnology and Bioengineering, Yuan Ze University, Jhongli, Taiwan; 2 Department of Horticulture and Biotechnology, Chinese Culture University, Taipei, Taiwan; 3 Institute of Plant Biology, National Taiwan University, Taipei, Taiwan; 4 Department of Life Science, National Taiwan Normal University, Taipei, Taiwan; 5 Department of Agronomy, National Chiayi University, Chiayi, Taiwan; 6 Department of Plant Medicine, National Chiayi University, Chiayi, Taiwan; 7 Department of Biochemical Science and Technology, National Chiayi University, Chiayi, Taiwan; 8 Department of Agricultural Chemistry, National Taiwan University, Taipei, Taiwan; Hainan University, CHINA

## Abstract

An abundant 17 kDa RNase, encoded by *OsPR10a* (also known as PBZ1), was purified from P_i_-starved rice suspension-cultured cells. Biochemical analysis showed that the range of optimal temperature for its RNase activity was 40–70°C and the optimum pH was 5.0. Disulfide bond formation and divalent metal ion Mg^2+^ were required for the RNase activity. The expression of *OsPR10a*::*GUS* in transgenic rice was induced upon phosphate (P_i_) starvation, wounding, infection by the pathogen *Xanthomonas oryzae* pv. *oryzae* (*Xoo*), leaf senescence, anther, style, the style-ovary junction, germinating embryo and shoot. We also provide first evidence in whole-plant system, demonstrated that *OsPR10a*-overexpressing in rice and *Arabidopsis* conferred significant level of enhanced resistance to infection by the pathogen *Xoo* and *Xanthomona campestris* pv. *campestris* (*Xcc*), respectively. Transgenic rice and *Arabidopsis* overexpressing *OsPR10a* significantly increased the length of primary root under phosphate deficiency (-P_i_) condition. These results showed that *OsPR10a* might play multiple roles in phosphate recycling in phosphate-starved cells and senescing leaves, and could improve resistance to pathogen infection and/or against chewing insect pests. It is possible that P_i_ acquisition or homeostasis is associated with plant disease resistance. Our findings suggest that gene regulation of *OsPR10a* could act as a good model system to unravel the mechanisms behind the correlation between P_i_ starvation and plant-pathogen interactions, and also provides a potential application in crops disease resistance.

## Introduction

In order to survive during phosphate (P_i_) starvation, plants have evolved the ability to increase the efficiency of P_i_ uptake via the up-regulation of a wide variety of intra- and extra-cellular ribonucleases (RNases) so as to scavenge and recycle P_i_ from organic phosphorus compounds [1]. For example, RNase LX is induced specifically in P_i_-limited tomato lateral and adventitious root primordia, suggesting that it might play a role in RNA turnover to supply P_i_ for root growth [2]. In tomato cells, P_i_ starvation has been found to induce three intracellular RNases (RNases LV 1–3) in vacuoles and one in the cytosol (RNase LX), and a secreted RNase (RNase LE) [3–5] was identified in culture medium. Moreover, secretomic analysis identified an extracellular ribonuclease 1 (RNS1) lacking the putative N-terminal signal peptide in P_i_-starved *Arabidopsis* suspension cells [6]. These studies indicated that ribonucleases play a key role in P_i_ scavenging and recycling from extra- and intra-cellular ribonucleic acids under phosphate starved (-P_i_) conditions.

Plant pathogenesis-related (PR) proteins are expressed in response to pathogen infection, environmental stresses and developmental processes, and some of them are expressed constitutively [7,8]. They can be divided into at least 17 different groups, PR-1 to -17, according to their amino acid sequences, immunological relationships and biological activities [9–11]. Among them, more than 100 PR-10-related genes have been reported from various plant species, and this PR protein family consists of acidic proteins with a low molecular weight (16–19 kDa) [12]. The PR-10 family has been identified as a group of intracellular proteins and their amino acid sequences show high similarity to the Bet v1-like superfamily, named after the white birch (*Betula verrucosa*) major pollen allergen [13–16]. Research has successfully identified at least five PR10-like genes/proteins in rice that respond to biotic stresses, such as pathogen infection [17–22], jasmonic acid (JA) and salicylic acid (SA) [23–25], or the expression of which are induced by abiotic stresses, including salt and drought [21,25,26]; the first identified gene/protein of this type was OsPR10a/PBZ1 [17]. Treatment of tobacco leaves and rice suspension-cultured cells with purified recombinant PBZ1 protein is known to be able to induce programmed cell death [27,28]. Furthermore, some PR-10 proteins are reported to possess *in vitro* ribonuclease activity, for example, PBZ1 (OsPR10a) and JIOsPR10 from rice (Kim et al. 2008b, 2011), LaPR10 from white lupin [29], CaPR-10 from hot pepper [30], ZmPR10.1 from maize [31] and SsPR10 from yellow-fruit nightshade [32], suggesting that the responses to biotic and abiotic stresses involving these proteins might be via their RNase activities in order to cope with environmental challenges. However, there have been only limited *in vivo* studies on the roles of PR10-related proteins in resistance to pathogen attack and the results remain to be verified.

In this study, we characterized an RNase, OsPR10a, and showed its inducible gene expression and enzyme activity in rice suspension-cultured cells under phosphate starvation. Ectopic expression of *OsPR10a*::*GUS* in rice demonstrated that *OsPR10a* was induced and expressed in P_i_-starved suspension-cultured cells, anther, style and the style-ovary junction, germinating embryo and shoot, senescing leaves or leaves that had been wounded or infected with a pathogen. Transgenic overexpression of *OsPR10a* under the control of the maize ubiquitin promoter (*Ubi*::*OsPR10a*) in rice and *Arabidopsis* plants showed the ability of OsPR10a to confer resistance to pathogen infection, and enhanced primary root growth under P_i_ deficiency condition.

## Materials and Methods

### Plant material and nutrient deprivation treatment

*Oryza sativa* L. cv Tainung 67 was used in this study. Immature seeds were de-hulled, sterilized with 2.4% NaOCl for 1 h, washed extensively with sterile water and placed on N6D agar medium for callus induction. After 1 month, callus derived from scutellum was transferred to liquid MS [33] complete medium (MS salts containing 3% sucrose and 10 μM 2,4-D (2,4-dichlorophenoxyacetic acid)) to establish a suspension cell culture. Suspension cells were cultured on a reciprocal shaker at 120 rpm and incubated at 26°C in the dark. Cells were subcultured routinely every week by transferring approximately 0.5 mL of cultured cells into 25 mL of fresh MS complete medium. In experiments carried out under conditions of phosphate, nitrogen and sugar starvation (referred to as -P_i_, -N and -S, respectively), KH_2_PO_4_, NH_4_NO_3_/KNO_3_ and sucrose were omitted from the MS complete medium, respectively. The cell weight and RNase activity were measured after 0, 1, 3, 5, 7 and 9 days of cultivation.

### Northern blot analysis

Total RNA was isolated from suspension-cultured cells using Trizol reagent (Invitrogen, Carlsbad, CA). RNA gel-blot analysis was performed as described previously [34]. Ten micrograms of total RNA were electrophoresed in a 1% agarose gel containing 10 mM sodium phosphate buffer (pH 6.5), transferred to a nylon filter and hybridized with a [α-^32^P]dCTP random-primer-labeled *OsPR10a* cDNA probe. The blot was visualized using autoradiography with X-ray film.

### Protein extraction and purification of the 17 kDa protein

Total proteins were extracted from suspension-cultured cells with an extraction buffer (50 mM Tris-HCl, pH 8.8, 1 mM EDTA, 10% [v/v] glycerol, 1% [v/v] Triton X-100, 10 mM β-mercaptoethanol and 0.1% [w/v] sarkosyl). Homogenates were centrifuged at 4°C and supernatants were concentrated by filtration with an Amicon Diaflo membrane PM10 (Amicon, Beverly, MA). The concentrated supernatant was subjected to continuous-elution electrophoresis. A Model 491 Prep Cell (Bio-Rad, Hercules, CA, USA) and a gel tube (3.7 × 8.0 cm) of 15% SDS-polyacrylamide were used for the electrophoresis. After 4 h, a total of 40 fractions were collected at 3-min intervals. These fractions were examined by 15% SDS-PAGE and subjected to an RNase activity assay. The fractions containing constituents with the expected molecular weight of 17 kDa were pooled and concentrated using Centricon devices (Amicon). The identity of the 17 kDa protein purified by gel tube electrophoresis was reconfirmed by an RNase activity assay.

### Proteins analysis by liquid chromatography/tandem mass spectrometry (LC/MS/MS)

The 17 kDa protein fraction from gel-filtration column chromatography fractionation was further separated by 12% SDS-PAGE with Coomassie blue staining. The gel of 17 kDa position was cut and then soaked in 25 mM NH_4_HCO_3_ for 10 min, and then incubated in 25 mM NH_4_HCO_3_ containing 50% acetonitrile for another 10 min, and then was subjected to in-gel tryptic digestion as descripted by Tsay et al. [35]. After digestion, the samples were purified and subjected to LC/MS/MS analysis. Amino acid sequence data processing was conducted as described by Tsay et al. [35,36].

### In-gel RNase activity assay

Total protein was extracted from rice suspension-cultured cells and medium was used as described above. Staining to identify the activity of RNase (EC 3.1.27.1) in 15% SDS-polyacrylamide gels was performed as described by Blank et al. [37] and Gallie et al. [38] with minor modifications. Separating gels containing 0.6 mg/mL yeast total RNA and 0.05 mg/mL bovine fibrinogen were subjected to electrophoresis. Then, SDS was removed by soaking the gel in 25% isopropanol containing 10 mM imidazole for 10 min, followed by incubation for another 10 min with 10 mM imidazole to remove isopropanol. Gels were incubated at 55°C in 100 mM imidazole, 200 mM KCl, 10 mM Tris-HCl pH 7.4 and 10 mM MgCl_2_ for 1 h, and washed for another 10 min with 10 mM imidazole to remove digested RNA fragments. Undigested RNA molecules remaining in the gel were stained with toluidine blue for 15 min, washed to remove excess dye, and clear bands indicating the positions of RNases were shown in the gel.

### RNase activity assay by spectrophotometry

Using yeast tRNA as a substrate, RNase activities were estimated from the release of ethanol-soluble free nucleotides by measuring absorbance at 260 nm, as described by Abel and Glund [39]. The enzyme unit (Wilson unit, WU) was defined as the amount of enzyme led to an increase in absorbance at 260 nm of 1.0 min^-1^ cm^-1^mL^-1^ [40].

### Expression and purification of OsPR10a protein

For the production of OsPR10a protein in *Escherichia coli*, the *OsPR10a* coding region was subcloned into *E*. *coli* expression vector pET-43.1a, which allowed a downstream in-frame fusion with the NusA-tag protein. The recombinant DNA was then transformed into *E*. *coli* BL21-DE3 host cells, followed by induction with IPTG, to produce NusA-tag/PR10a fusion protein. The crude extract of total protein obtained from bacterial cells was passed through a nickel affinity column (Ni^2+^-NTA, BD) to isolate the NusA-tag/PR10a recombinant proteins, in accordance with the manufacturer’s protocol. After washing the column with equilibration buffer, the protein-bound resin was eluted with a linear gradient of 0–0.5 M imidazole. The fractions containing NusA-tag/PR10a were pooled, dialyzed and concentrated by ultrafiltration (Amicon Centriprep). The purified recombinant proteins were then digested with enterokinase for 16 h at room temperature, and this reaction solution was subjected to another round of purification with a Ni^2+^-NTA Sepharose column to remove the NusA-tag. The flow-through containing the cleaved OsPR10a protein was collected by centrifugation and the desired cleavage products of OsPR10a were separated from the NusA-tag.

### RNase activity analysis of recombinant OsPR10a protein

To examine the RNase activity of recombinant OsPR10a isolated from *E*. *coli*, reducing agents including 5% β-mercaptoethanol (β-ME) and 10 mM dithiothreitol (DTT), and a divalent cation chelator EDTA (10 mM) were tested. Reaction buffer (25 mM sodium acetate, pH 5.0; 2 mM MgCl_2_) containing 10 μg of rice total RNA was supplemented with each of the above-mentioned reagents, respectively, followed by incubation with or without 1.0 μg of recombinant OsPR10a protein at 37°C for 2 h. RNase activity was determined by the level of degradation of rice total RNA determined by agarose gel electrophoresis.

### Primers

The sequences of all primers used for PCR and RT-PCR amplification are listed in S1 Table.

### Construction of expression vectors

To construct the *OsPR10a*::*GUS* expression vector, a 1.4-kb DNA fragment containing the promoter and 5′-untranslated region of *OsPR10a* (S1 Fig) was PCR-amplified using the primers *OsPR10aP-5P* and *OsPR10aP-3B*. This DNA fragment was cleaved with *Pst*I and *Bam*HI, and cloned into vector pBX-2 as described previously [34]. To construct a vector (*Ubi*::*OsPR10a*) for the ectopic expression of *OsPR10a* in transgenic *Arabidopsis*, a 479-bp DNA fragment that contained the complete coding region of *OsPR10a* (S1 Fig) was amplified using primers *OsPR10a-5P* and *OsPR10a-3P*. This DNA fragment was digested with *Pst*I and cloned into the *Pst*I site of expression vector pAHC18 [41] under the control of the maize ubiquitin promoter. Both *OsPR10a*::*GUS* and *Ubi*::*OsPR10a* constructs were linearized by digestion with *Hin*dIII and inserted into the *Hin*dIII site of the pSMY1H binary vector [34], respectively, followed by *Agrobacterium*-mediated gene transformation.

### Plant transformation and RT-PCR

Plasmids were introduced into *Agrobacterium tumefaciens* strain EHA105 [42] by electroporation, and rice calli were transformed as described previously [34]. For the ectopic expression of *OsPR10a* in *Arabidopsis* plants, the binary plasmid constructs were introduced into *Arabidopsis* plants using *A*. *tumefaciens* strain GV3101 by the floral dip method [43]. The T1, T2 and T3 transformed plants were selected on ½ MS medium [33] containing 30 mg/L hygromycin B. The expression levels of *OsPR10a* in *Arabidopsis* were evaluated by RT-PCR and total RNA isolated from leaves of 7-day-old seedlings was used to synthesize first-strand *cDNAs using oligo(dT) primers*, *which were subjected to PCR analyses using the primers OsPR10a-RT5* and *OsPR10a-RT3*. PCR products were resolved by agarose gel electrophoresis and visualized by ethidium bromide staining.

### Histochemical staining for GUS activity in rice leaves

For GUS activity staining of rice cells, the suspension-cultured cells were subjected to phosphate starvation or inoculated with *Xanthomonas oryzae* pv. *oryzae* (*Xoo*) (1.0×10^6^ CFU/mL) for 1 day. For GUS staining of rice leaves, three-leaves-stage of rice seedlings were wounded mechanically with a razor blade, or infected by spraying with 5 mL *Xoo* (1.0×10^8^ CFU/mL) per plant, and growth in 25°C culture room with relative humidity above 70% under a 16 h/8 h light and dark cycles for 2 days. After treatments, the rice cells and the second leaves from treated plants were isolated and incubated in 1 mM 5-bromo-4-chloro-3-indolyl β-D-glucuronide (X-gluc) reaction solution (in 100 mM sodium phosphate, pH 7.0, 10 mM EDTA, 0.5 mM potassium ferrocyanide, 0.5 mM potassium ferricyanide, 0.1% Triton X-100) at 37°C in the dark for 24 h. Then, leaves were decolorized in 70% ethanol at 37°C for 24 h. The stained cells and leaves were preserved in 70% ethanol and rinsed with water before being photographed.

### Pathogen inoculation in rice and *Arabidopsis*

For leaf infiltration with a pathogen in *Arabidopsis*, two leaves per plant from the wild type (WT) and three independent transgenic lines were injected with the bacteriumaonsider: in thed in Pi nsistent in this regard main text. *X*. *campestris* pv. *campestris* (*Xcc*) (1.0×10^8^ CFU/mL) were inoculated at four sites per leaf using a syringe. For *Xoo* inoculation of rice plants, rice leaf tips of 4-week-old plants were wounded at five sites by a *Xoo*-contaminated needle followed by sprayed with *Xoo* (1.0×10^8^/mL, 3.0 mL/pot) once per day for three times as previous description by Nakashita et al. [44] and Xu et al. [45] with minor modification. Inoculated plants were kept in a growth chamber (90% relative humidity, 28°C and 16-h photoperiod) for the development of disease symptoms. For inoculation of *Xcc* in whole-plant of *Arabidopsis*, experiments were performed as described previously by Simpson and Johnson [46] with minor modifications. One pot containing three 4-week-old plants were sprayed with a 1.5 mL of *Xcc* bacterial suspension (1.0×10^8^ CFU/mL). Inoculated plants were kept in a growth chamber (80–90% relative humidity, 23°C and 16-h photoperiod) for the development of disease symptoms. Five days after inoculation of *Arabidopsis*, disease symptoms on leaves were photographed and evaluated by measuring the area of the necrotic lesions.

### Hydroponic culture of rice

Rice seeds were sterilized by 2.4% NaOCl and germinated in water for 3 days at 28°C in the dark. Emerged seeds with 2 mm length of primary roots were selected and then placed onto a net floating in a half-strength of Kimura B solution [47] either with 12.4 mg/L KH_2_PO_4_ or not, respectively. The cultured medium was refreshed every 3 days. Plants were grown in a growth chamber at 28°C under 16 h light and 8 h dark photoperiod for 12 d. Both root length and the plant height of rice seedlings were measured and then photograph after treatments.

### Phosphate starvation treatment of rice and *Arabidopsis*

For *Arabidopsis*, seeds were sterilized and sown on vertical plates containing solid half-strength of MS medium containing15 g/L sucrose either without (-P_i_) or with (+P_i_) 85 mg/L KH_2_PO_4_, respectively, and incubated in 22°C for 7–10 days under 16 light/ 8 dark photoperiods, and the lengths of roots were measured and photographed. For rice, seeds were sterilized and imbibed in distilled water for 3 days, germinating embryos were isolated and placed onto the vertical plates containing solid half-strength of MS medium including 15 g/L sucrose either without (-P_i_) or with (+P_i_) 85 mg/L KH_2_PO_4_, respectively, and then were incubated for another 5–10 days. The lengths of roots and shoots were measured and photographed.

## Results

### Effect of nutritional stresses on the RNase activities in rice suspension-cultured cells

**Fig 1:**
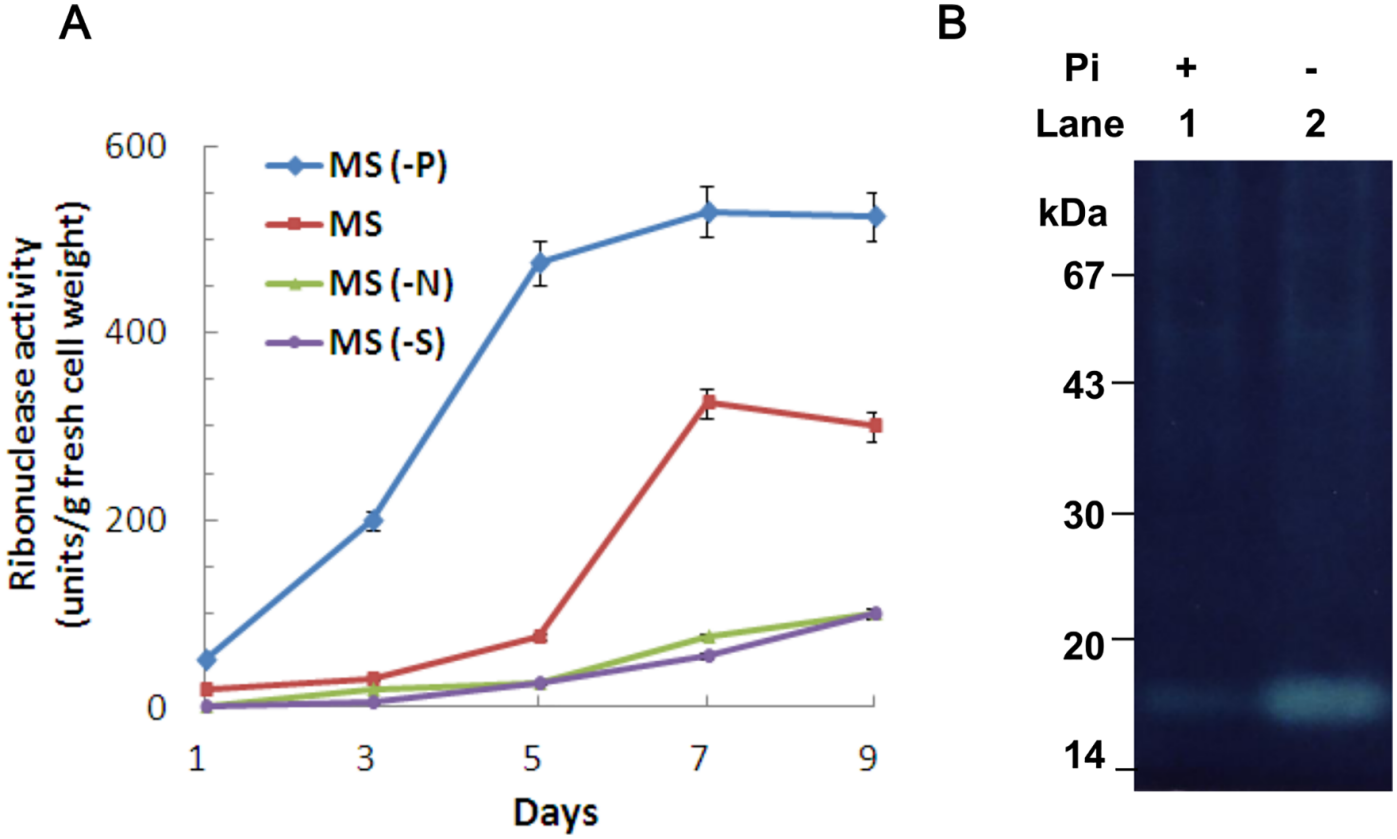
RNase activities in rice suspension-cultured cells. (A) Rice cells were suspension-cultured in MS complete medium (containing 3% sucrose and 10 μM 2,4-D) for 3 days then transferred to MS complete medium with (■) or without (●) sucrose, or deficient in either phosphate (♦) or nitrogen (▲), and cells were collected at the time points indicated on the x-axis. Error bars indicate standard errors for the measurements from at least three individual experiments. (B) Rice suspension cells were grown for 3 days in MS medium with (+P_i_) or without (-P_i_) phosphate. Total protein was extracted from cultured cells and 40 μg of total cellular protein were applied to an in-gel RNase activity assay.

To study the effects of various nutrient deficiencies on the growth of suspension-cultured rice cells, cells were grown in MS complete medium (MS), or MS medium deficient in phosphate (-P_i_), nitrogen (-N) or sucrose (-S). Suspension-cultured rice cells were collected at 1-, 3-, 5-, 7- and 9-day intervals. As shown in S2 Fig, at over 7 days of cultivation, a large increase of fresh cell weight occurred for cells cultured in MS medium; there was a moderate increase in -P_i_ medium, a slight increase in -N medium and a slow decrease in -S medium. It is known that phosphate starvation can induce various activities of cellular RNases [48], so we checked whether RNase activities were induced in cultured rice cells under conditions with a limitation of P_i_ or the other nutrients. When cells were cultured in -P_i_ medium (Fig 1A), a rapid increase in RNase activity was observed, which peaked on day 5 and was then maintained at a plateau until day 9. However, there was only a slight increase in RNase activity from day 1 to day 5 in complete MS, but a dramatic increase was observed on day 7. In contrast, RNase activities in both -N and -S media were barely detectable during the first 5 days of incubation, and then increased gradually from day 7 onwards. To further study the P_i_-starvation-induced RNase in rice cells, suspension-cultured cells were grown in +P_i_ and -P_i_ liquid media for 5 days and total proteins were purified from the cultured cells, followed by an in-gel RNase activity assay. In a gel containing yeast t-RNA, a clear zone at around 17 kDa was detected in both +P_i_- and -P_i_-treated cells (Fig 1B), but much higher activity was observed in protein extracts isolated from -P_i_ than +P_i_-treated cells, indicating that the RNase activity was induced under P_i_ starvation.

### Purification of the 17 kDa RNase

**Fig 2:**
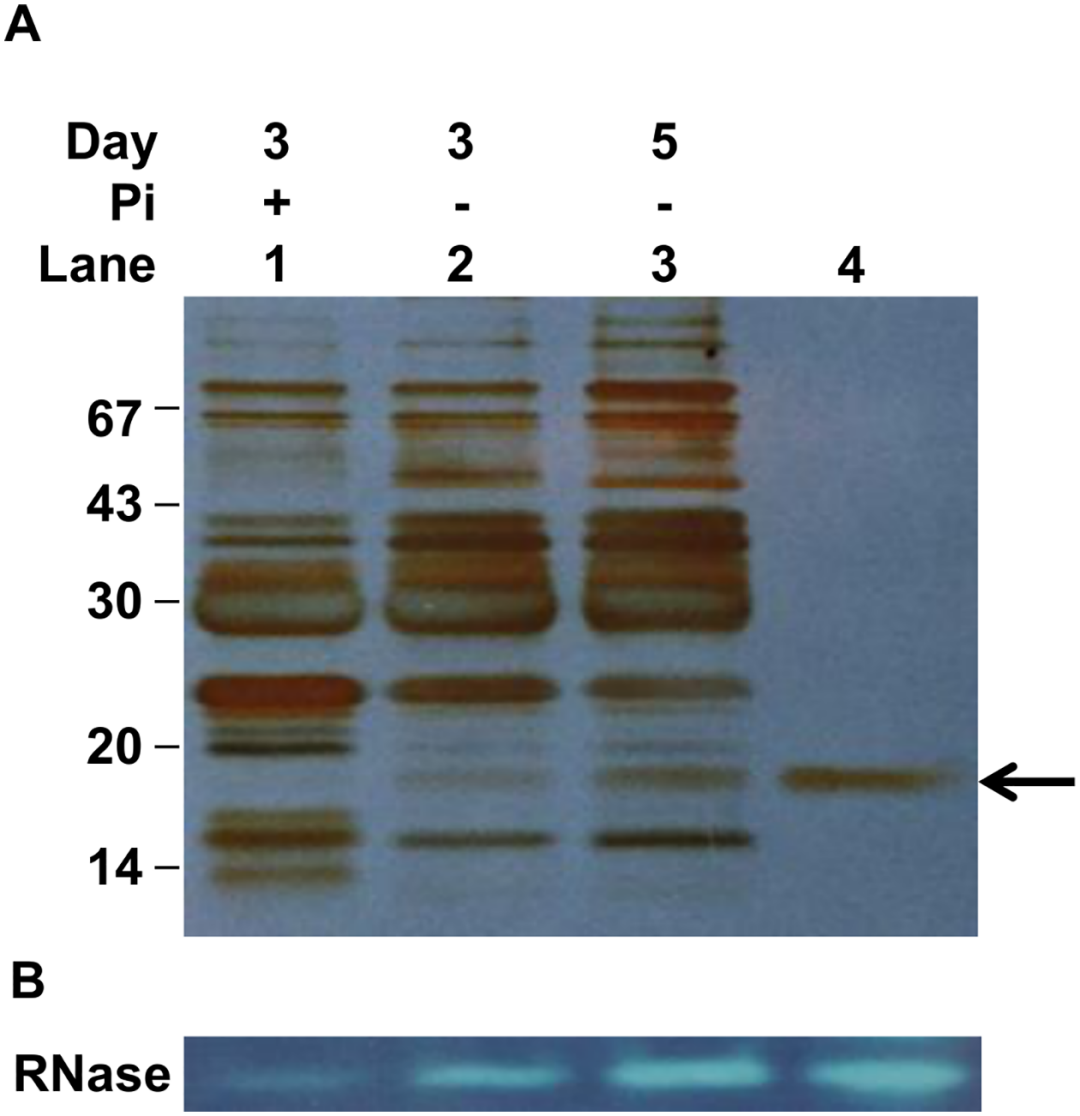
Purification of the 17 kDa protein induced by phosphate starvation and its RNase activity assay. Total crude extracted proteins were purified from rice suspension cells that were cultured in either MS medium (lane 1) or MS without phosphate for 3 days (lane 2) or 5 days (lane 3). Total protein derived from phosphate-starved cells was collected and subjected to purification of the 17 kDa protein by continuous-elution electrophoresis (lane 4). A total of 5 μg of crude extracted proteins and 0.5 μg of purified proteins were subjected to SDS-PAGE followed by silver staining (A) or in-gel RNase activity assay (B). Arrow indicates the position of 17 kDa.

To isolate and identify the 17 kDa RNase, rice cells were grown in MS liquid medium for 3 days, followed by P_i_ starvation treatments for 3 and 5 days. Total proteins were extracted from cultured cells and subjected to SDS-PAGE analysis followed by silver staining. Two induced proteins appeared at positions corresponding to sizes of about 17 and 48 kDa under P_i_ starvation conditions (Fig 2A, lanes 1–3). We proposed that the P_i_-starvation-induced 17 kDa protein might be the protein associated with the P_i_-starvation-induced 17 kDa RNase activity shown in Fig 1. To examine whether the 17 kDa protein had RNase activity, protein purification was conducted by gel-filtration column chromatography fractionation and a 17 kDa single-protein fraction was obtained (Fig 2A, lane 4). A duplicate gel was subjected to an in-gel RNase activity assay. The RNase activity was detectable at the same position as the 17 kDa protein band, and was induced under P_i_ starvation conditions (Fig 2B, lanes 1–3). The changes in the amount of the 17 kDa protein were consistent with the RNase activity levels with an increasing number of days of P_i_ starvation (compare Fig 2A and 2B, lanes 1–3). Furthermore, the purified protein at the same position on gels displayed RNase activity (Fig 2B, lane 4). These results demonstrate that the purified 17 kDa protein possessed RNase activity, and both the levels and the activities of the protein were upregulated in P_i_-starved rice cells.

**Fig 3:**
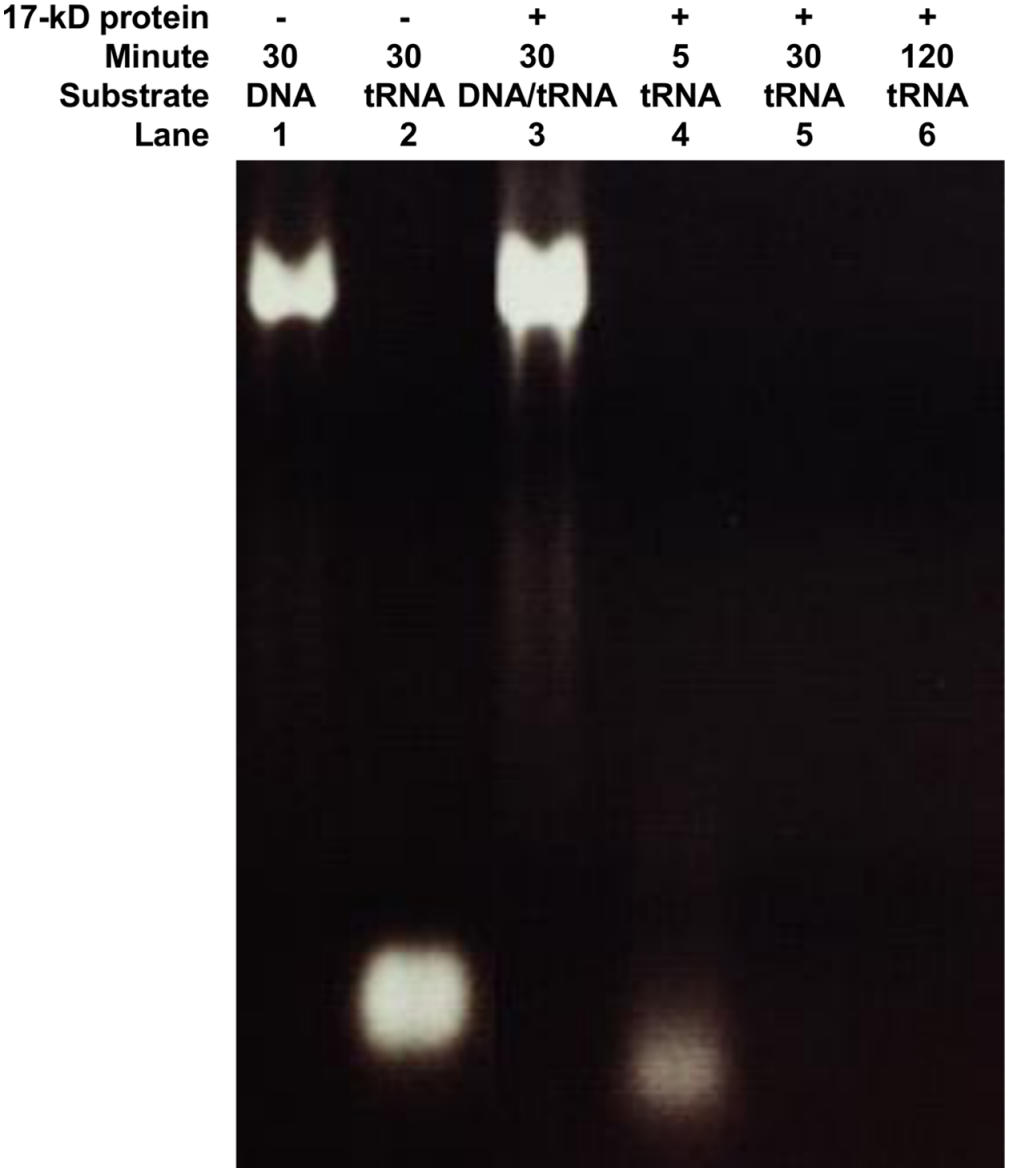
Agarose gel electrophoresis of enzymatic hydrolysates of yeast tRNA by the purified 17 kDa protein. A total of 0.5 μg of purified 17 kDa protein was mixed with or without 1.0 μg of rice genomic DNA (DNA) or 5.0 μg of yeast t-RNA (tRNA). The mixtures were incubated for the indicated times followed by 1.5% agarose gel electrophoresis.

Yeast tRNA and/or rice genomic DNA were further applied as substrates to analyze the RNase activities of the 17 kDa protein. Rice genomic DNA was insensitive to the 17 kDa protein (Fig 3, lane 3), whereas yeast tRNA was partially degraded at 5 min (Fig 3, lane 4) and was digested completely after 30 and 120 min of incubation (Fig 3, lanes 5–6) in the presence of the 17 kDa protein, suggesting that the 17 kDa protein has ribonuclease activity that can cleave RNA rather than genomic DNA.

### RNase activity assay of the 17 kDa protein

**Fig 4:**
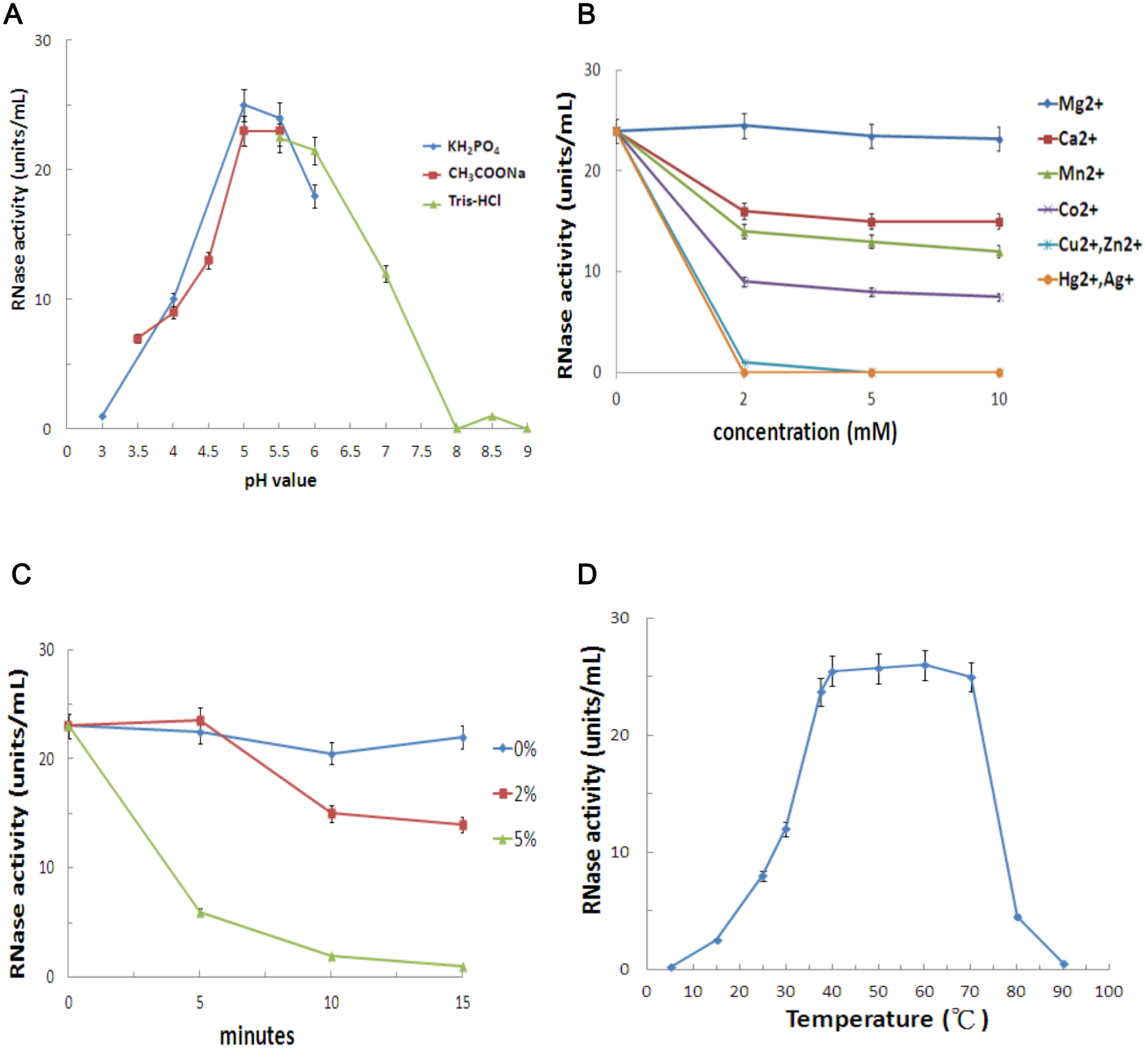
Physical and biochemical properties of RNase activity of 17 kDa protein. The 17 kDa protein was treated with (A) different levels of acidity (pH) in potassium phosphate, sodium acetate or Tris-HCl buffers; (B) metal ions including Mg^2+^, Ca^2+^, Mn^2+^, Co^2+^, Cu^2+^, Zn^2+^, Hg^2+^ and Ag^+^ at different concentrations (0–10 mM); (C) different concentrations (0%, 2% and 5%) of β-mercaptoethanol for different times (0–15 min); and (D) heating at different temperatures from 5 to 90°C under pH 5.0. RNase activity assay was conducted using a spectrophotometer, as described by Abel and Glund [39]. Error bars indicate standard errors for the measurements from at least three individual experiments.

To characterize the RNase activity of the 17 kDa protein, the influence of factors including pH, metal ions, the reducing agent β-mercaptoethanol (β-ME) and temperature were tested. Changes in RNase activity across various ranges of pH were monitored using potassium phosphate (pH 3.0–6.0), sodium acetate (pH 3.5–5.5) and Tris-HCl (pH 5.5–9.0) buffers. The optimal pH of RNase activity for this 17 kDa protein was pH 5.0 (Fig 4A). Moreover, shifts to a pH level either more acidic or more basic than pH 5.0–6.0 resulted in sharp declines in RNase activity and we observed complete loss of activity at pH 3.0 and 8.0. To examine the effects of various metal ions on RNase activity, 1.0 μg of purified 17 kDa protein was mixed with reaction buffer (25 mM sodium acetate, pH 5.0), containing Mg^2+^, Ca^2+^, Mn^2+^, Co^2+^, Cu^2+^, Zn^2+^, Hg^2+^ and Ag^+^ at different concentration (0–10 mM). RNase activities were detected following incubation at 37°C for 30 min. RNase activities did not show any significant changes by Mg^2+^, but partially inhibited by Ca^2+^, Mn^2+^ and Co^2+^, whereas almost completely inhibited by Cu^2+^, Zn^2+^, Hg^2+^ and Ag^+^ (Fig 4B). These results suggest that Mg^2+^ might be required to maintain RNase activity, we therefore use the solution containing 2 mM MgCl_2_ and 25 mM sodium acetate at pH 5.0 as a reaction buffer for further RNase activity studies. We next examined the effect of the reducing agent β-ME on RNase activity; 1.0 μg of the purified 17 kDa protein was treated with reaction buffer containing 0%, 2% or 5% β-ME. After 10 min of incubation, 2% β-ME partially impaired the RNase activity, and after 5 min of 5% β-ME treatment, the RNase activity was severely reduced, while it was almost completely lost after 10 min of incubation (Fig 4C). These results indicated that disulfide bonds were necessary for the 17 kDa protein to display its RNase activity. To determine the optimum temperature for RNase activity of the 17 kDa protein, reaction buffer containing 1.0 μg of 17 kDa protein was incubated for 10 min at temperatures from 5°C to 90°C. The RNase activity abruptly declined when the incubation temperature was lower than 40°C or higher than 70°C, suggesting a wide temperature range (40–70°C) in which the RNase activity of this 17 kDa protein is optimal (Fig 4D).

### The 17 kDa protein is identified as the rice OsPR10a (PBZ1) protein and its gene expression was induced under phosphate starvation

**Fig 5:**
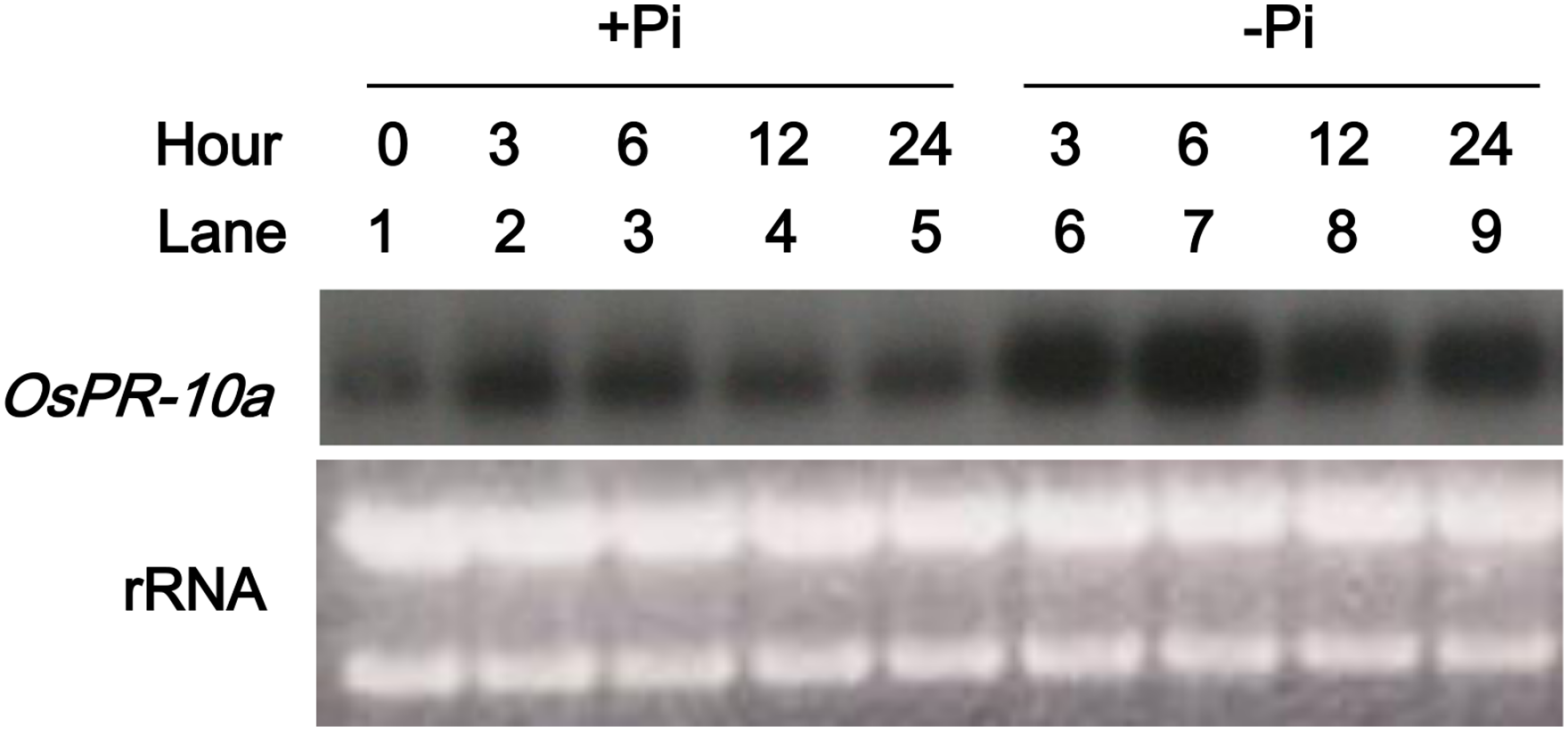
Phosphate starvation induced *OsPR10a* gene expression in rice suspension-cultured cells. Rice suspension cells were cultured in MS medium for 3 days and transferred to MS medium supplemented with (+P_i_) or without (-P_i_) phosphate. Total RNA was isolated from cells and subjected to northern blot analysis using the *OsPR10a* coding region as a probe. The rRNA served as a total RNA loading control.

To make protein identification, the 17 kDa protein isolated from gel-filtration column chromatography fractionation (Fig 2A, lane 4) was subjected to the LC/MS/MS analysis. Six protein candidates were obtained and used as a query for a Blast search as described in materials and methods. Among which only one candidate protein, D38170, predicted to have enzymatic ribonuclease activity is the probenazole-inducible protein 1 (PBZ1/OsPR10a) [17]. Rice *OsPR10a* encodes a peptide of 158 amino acid residues with a predicted molecular mass of 16.7 kDa, which is very similar to the size of the 17 kDa protein that we had studied. To verify that OsPR10a was our target protein and that it exhibits a P_i_-starvation-inducible gene expression pattern, rice cells were cultured in MS (+P_i_) or MS without P_i_ (-P_i_) medium for various periods, and total RNAs isolated from these cell samples were subjected to gel-blot analysis using *OsPR10a* cDNA as a probe. The accumulation of *OsPR10a* mRNA was much greater in P_i_-starved cells than in cells cultured on P_i_-containing MS medium (Fig 5), which demonstrated that the expression of *OsPR10a* was stimulated by P_i_ starvation, but suppressed in P_i_-rich cells. The expression pattern of *OsPR10a* paralleled the results obtained in the in-gel RNase activity assays, as shown in Figs 1B and 2B, which confirmed that the 17 kDa protein observed there was OsPR10a.

### OsPR10a has ribonuclease activity

To study the RNase activity of rice OsPR10a further, production of OsPR10a recombinant proteins was conducted as described in the Methods section. The resulting NusA-tag/OsPR10a recombinant proteins were analyzed by SDS-PAGE, followed by silver staining, results showed that the major product of NusA-tag/OsPR10a recombinant protein, which was consistent with the predicted molecular mass of 83 kDa (66 kDa for NusA-tag and 17 kDa for OsPR10a) (S3A Fig, lanes 1 and 2, bold arrow). The purified fusion protein was then digested with enterokinase for 16 h at room temperature, and the reaction solution was subjected to another round of purification with a Ni^2+^-NTA Sepharose column to remove the NusA-tag. The desired cleavage products of the 17 kDa OsPR10a (S3A Fig, lane 3, asterisk) and the 66 kDa NusA-tag (S3A Fig, lane 4, dashed arrow) were observed. To examine the RNase activity of OsPR10a produced in *E*. *coli*, reaction buffers containing either reducing or chelating agents were tested using rice total RNA as a substrate. The degradation of rice RNA was only shown in reaction buffer containing the purified OsPR10a protein (S3B Fig, lane 1). There was no significant RNA degradation when rice RNA was treated with OsPR10a supplemented with either β-ME, DTT or EDTA. Furthermore, the degradation of rice total RNA increased progressively with an increase of the incubation time during which the reaction buffer was supplemented with bacterially purified OsPR10a (S3C Fig) in the absence of reducing or chelating agents. These results reconfirmed that OsPR10a possesses RNase activity.

### Regulation of *OsPR10a* expression by multiple modalities

**Fig 6:**
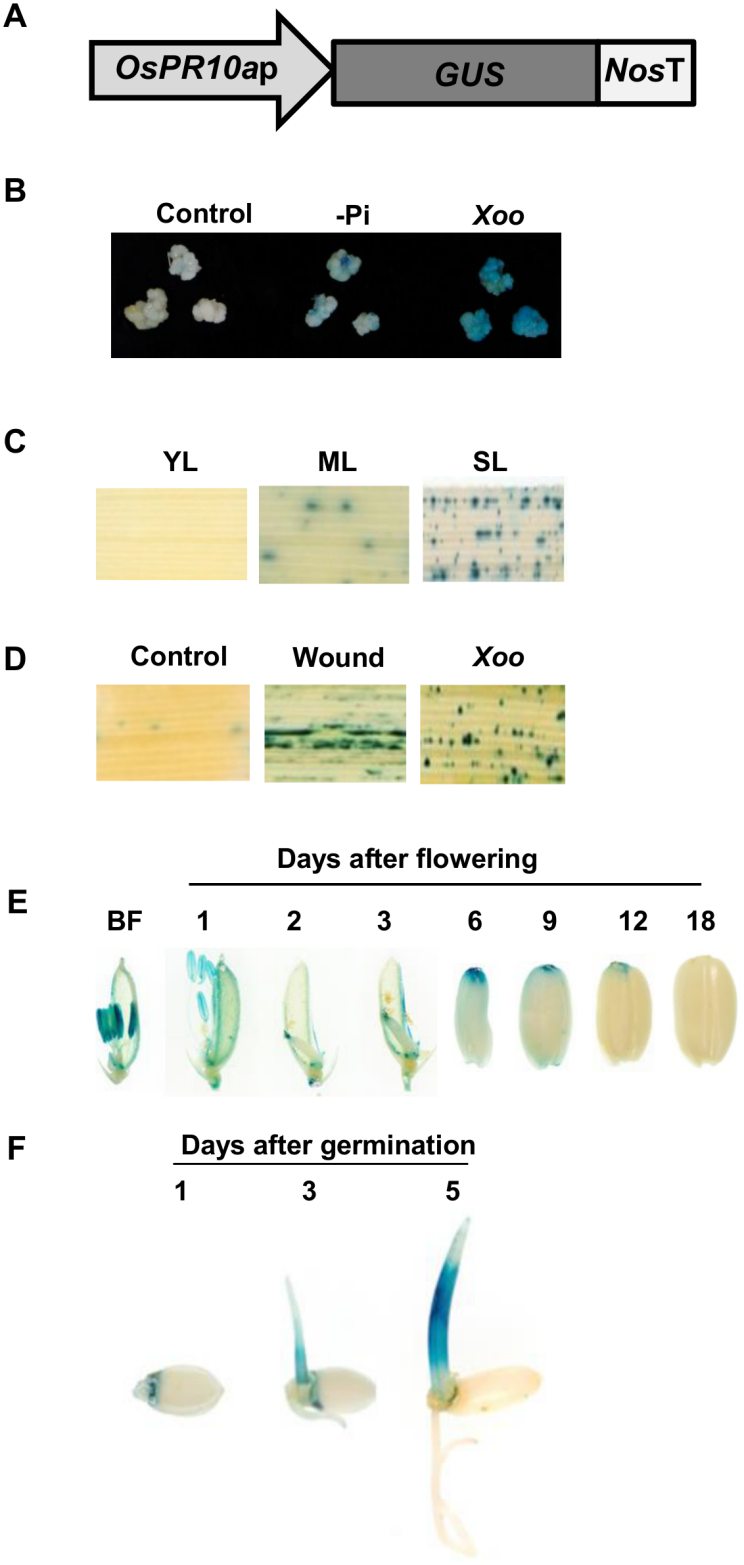
Histochemical staining for β-glucuronidase (GUS) activity in suspension-cultured cells, leaves, flowers, developing and germinating seeds of transgenic plants. (A) Schematic diagram of the *OsPR10a*::*GUS* expression construct. (B) GUS staining in rice suspension-cultured cells under phosphate starvation or following *Xanthomonas oryzae* pv. *oryzae* (*Xoo*) inoculation. (C) GUS expression patterns in young leaf (YL), mature leaf (ML) and senescent leaf (SL). (D) GUS staining in leaves with no treatment (control), wounding (Wound) or after spraying with *Xoo*. (E) GUS staining in flowers and developing seeds. BF: before flowering. (F) GUS staining in germinating seeds.

To determine the *OsPR10a* expression profile in rice, we generated *OsPR10a*::*GUS* transgenic lines expressing a GUS reporter gene under the control of the 1.44-kb *OsPR10a* promoter (from -1340 to +100; S1 Fig) (Fig 6A). In suspension-cultured cells, GUS expression was moderately induced in -P_i_ medium, but strongly activated when the culture medium contained the pathogen *X*. *oryzae* pv. *oryzae* (*Xoo*), which causes bacterial blight disease in rice (Fig 6B). GUS expression was not detectable in young growing rice leaves, but low levels were observed in mature leaves and there was strong activation in senescing leaves (Fig 6C). When mature leaves were mechanically wounded with a razor blade, strong GUS expression was restricted to the wounded region, but was weakly induced in the uncut region in the same leaves. GUS activity was also detectable in mature leaves after 5 days of inoculation with *Xoo* (1.0×10^8^ CFU/mL) by spraying (Fig 6D).

We also examined *OsPR10a*::*GUS* expressing in flowers and developing seeds (Fig 6E). Before flowering, strong GUS staining activity was observed in anthers but a weak signal in style and lemma. Right after flowering, GUS activity was significantly detected in lemma, anthers, style and the style-ovary junction, but not in filament, stigma and ovary. After 2–3 days of flowering, GUS activity was still high in the upper region of ovary (developing seeds) which below the remaining style, and a weak staining was seen in lemma, and GUS staining signals were gradually disappeared after 6–18 days of flowering. In germinating seeds, the expression of *OsPR10a*::*GUS* was specifically in embryos and shoots, but not in roots and endosperms (Fig 6F).

### Overexpression of O*sPR10a* confers resistance against *Xoo* infection in rice

**Fig 7:**
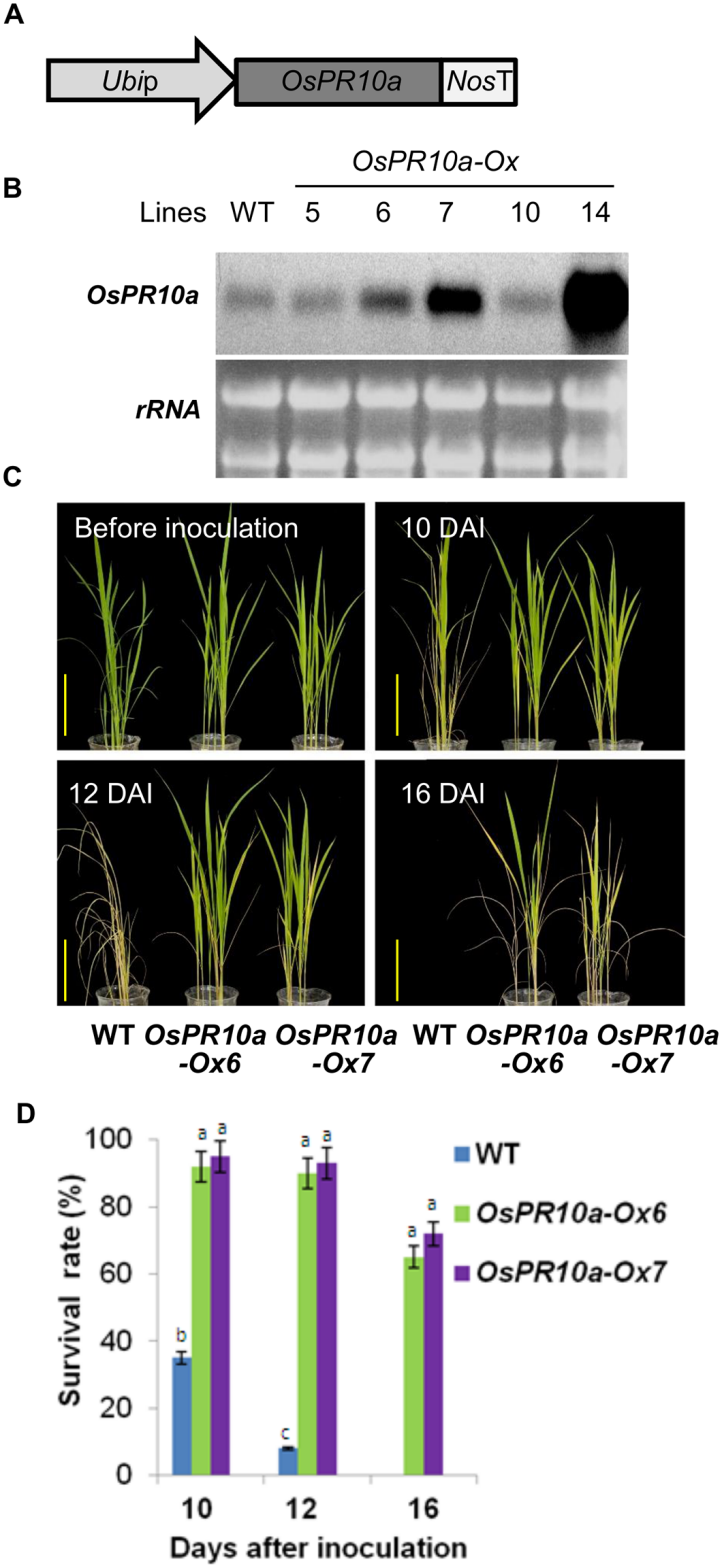
Overexpression of *OsPR10a* in rice enhanced resistance to *Xoo* infection. (A) Schematic diagram of the *Ubi*::*OsPR10a* overexpression construct. *Ubip*: the maize *ubiquitin* promoter; *OsPR10a*: the coding sequence of *OsPR10a*; *NosT*: nopaline synthase terminator. (B) The expression of *OsPR10a* mRNA in four independent transgenic lines determined by northern-blot analysis using *OsPR10a* coding region as a probe. (C) Ectopic expression of *OsPR10a* in rice enhanced resistance to *Xoo* infection. 4-week-old plants (4–5 plants per pot) were wounded in leaf tips and sprayed with *Xoo* (1.0×10^8^ CFU/mL). Photographs were taken at 0, 10, 12 and 16 days after inoculation. Bars = 10 cm. After 16 DAI, all WT plants (100%) were dead and collapsed, therefore they were not photographed. (D) Quantification of the plants that survived after *Xoo* infection. Four-week-old rice plants were sprayed with *Xoo* (1.0×10^8^ CFU/mL) as described above. The experiments were repeated three times. Groups that share the same letter are not significantly different estimated by ANOVA (P 0.05). Data are shown as means ±SD (n = 10). DAI: days after inoculation.

Although the role of *OsPR10a* has considered as disease-related function in rice, but evidence from the in vivo studies are still limited and need to be ascertained. Therefore, we next would like to examine whether overexpression of *OsPR10a* was associated with able to enhance rice resistance against *Xoo*. The expression cassette (*Ubi*::*OsPR10a*) that containing *OsPR10a* coding region under the control of maize *ubiquitin* promoter was constructed (Fig 7A) and the transgenic plants were generated by *Agrobacterium*-mediated gene transformation. Twelve independent transgenic lines were obtained, and six independent lines carried single T-DNA insertion were detected by Southern blot analysis (S4 Fig). Among them five transgenic lines were randomly selected for northern blot analysis. When compared with the WT plants, the abundance of *OsPR10a* mRNA levels was increased slightly in line 6, but moderately in line 7 and strongly in line 14, whereas no significant difference in lines 5 and 10 (Fig 7B). In order to eliminate functional side effects result from the highly transgene-overexpressing plant [49], the transgene *OsPR10a* which displayed moderate expression in transgenic line 6 (*OsPR10a-Ox6*) and line 7 (*OsPR10a-Ox7*) were therefore selected for antibacterial activity assay. Four-week-old of rice leave tips of both transgenic lines and WT plants were wounded by a *Xoo*-contaminated needle, and were followed by sprayed on whole-plants with *Xoo* (1.0×10^8^ CFU/mL) once per day for three times and then were grown in 28°C culture room with relative humidity above 90% under a 16 h/8 h light and dark cycles. Before *Xoo* inoculation, there were no significant differences in plant phenotypes among these three tested lines (Fig 7C, upper left). However, after 10 days of *Xoo* inoculation (10 DAI), a severe of disease symptoms were observed in WT plants, among which up to 65% of plant leaves turning brown, and later wilted and dead, whereas only a few *OsPR10a* overexpressing plants displaying slightly chlorotic symptoms in the first leaves. After 12 DAI, almost of the all WT plants (92%) were wilted and standing dead, but only a few leaves displaying chlorotic symptoms in *OsOR10a* overexpressing plants. After 16 DAI, all WT plants (100%) were dead and collapsed, whereas the both transgenic lines showed significant resistance to *Xoo* infection, in which only some of plant leaves displaying brown/yellow chlorosis symptoms, and only a few dead plants were observed. After 16 days of *Xoo* inoculation, the survival rates of WT, *OsPR10a-Ox6* and–*Ox7* were 0%, 65% and 72%, respectively (Fig 7D). Moreover, the expression of three defense-related genes [11,50], *OsPR1*, *OsPR4*, and *OsPR10e*, in the *OsPR10a*-overexpressing lines was analyzed to further determine the effect of *OsPR10a* on pathogen defense-related genes expression. Analysis of *Xoo* infected leaves indicated that the respective abundances of *OsPR1*, *OsPR4*, *and OsPR10e* mRNAs in *OsPR10a*-overexpressing lines were no significant difference as compared with those of wild type (S5 Fig).

### Expression of *OsPR10a* enhanced *Arabidopsis* tolerance to pathogen *Xanthomonas campestris pv. campestris (Xcc)* infection

**Fig 8:**
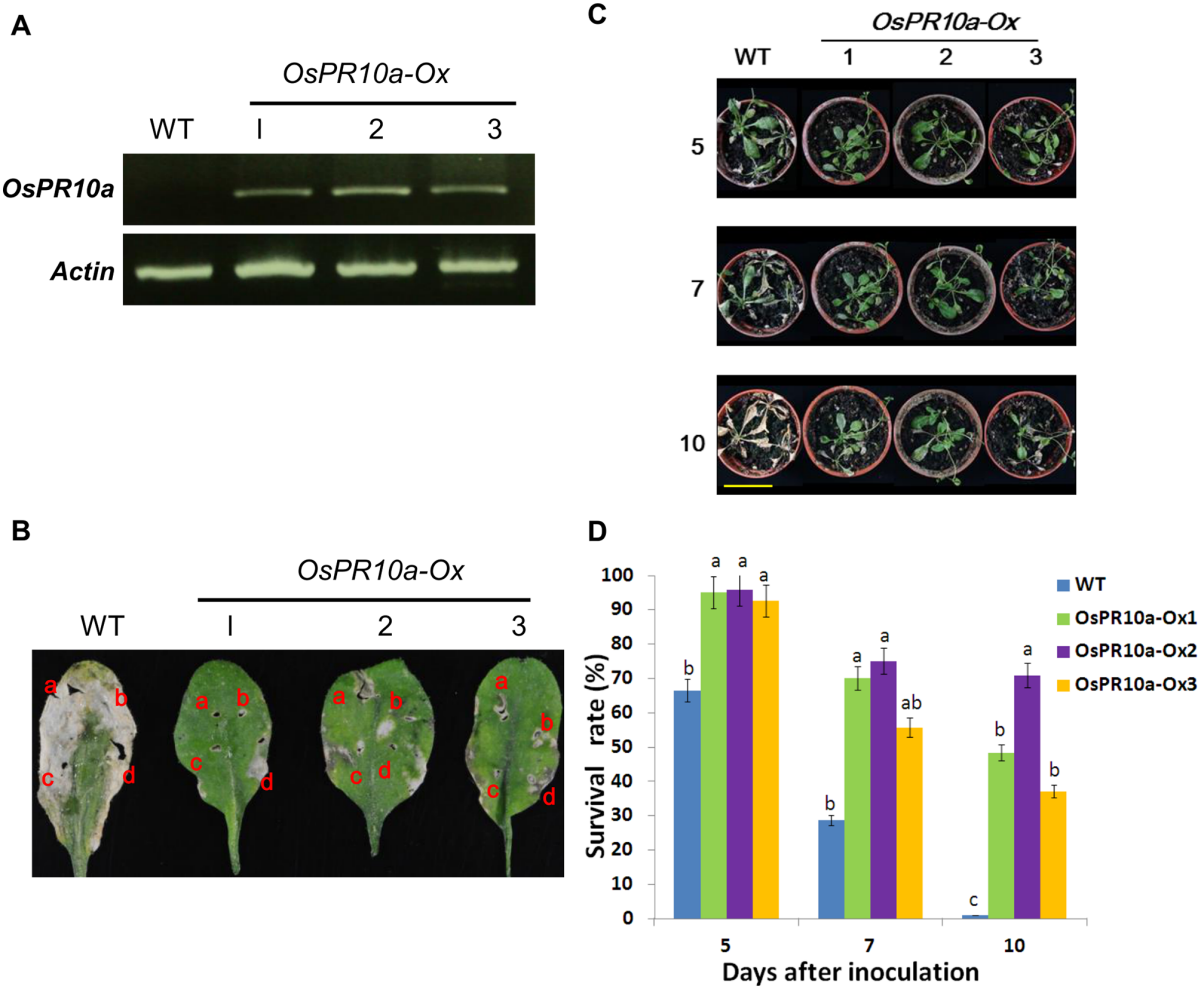
Overexpression of *OsPR10a* in *Arabidopsis* enhanced resistance to *Xanthomonas campestris pv*. *campestris* (*Xcc*) infection. (A) The expression of *OsPR10a* mRNA in three independent transgenic *Arabidopsis* lines determined by RT-PCR. (B) Resistance to *Xcc* on leaves. Healthy leaves from 4-week-old soil-grown wild-type (WT) and *OsPR10a*-overexpressing plants were inoculated with *Xcc* (1.0×10^8^ CFU/mL) using a syringe. This photograph was taken 5 days post-inoculation. (C) Ectopic expression of *OsPR10a* in *Arabidopsis* enhanced resistance to *Xcc* infection. 4-week-old plants were sprayed with *Xcc* (1.0×10^8^ CFU/mL). Photographs were taken at 5, 7 and 10 days after inoculation. The necrosis in WT plants was initially visible 3 to 5 days after inoculation, and death of the whole plant occurred by 10 days. Bars = 5 cm. (D) Quantification of the plants that survived *Xcc* infection. Four-week-old *Arabidopsis* plants were sprayed with *Xcc* (1.0×10^8^ CFU/mL). The experiments were repeated three times. Groups that share the same letter are not significantly different estimated by ANOVA (P 0.05). Data are shown as means ±SD (n = 10).

To determine whether *OsPR10a* also plays a role in disease resistance in dicot plants such as *Arabidopsis*. 20 transgenic *Arabidopsis* plants containing the expression cassette *Ubi*::*OsPR10a* (Fig 7A) were generated, and three of them were randomly selected, designated as *OsPR10a-Ox*-1, -2 and -3, for antibacterial activity assays. RT-PCR analysis showed that the transgene *OsPR10a* was expressed in each transgenic line, but not in WT plants (Fig 8A). To examine pathogen resistance, two leaves per plant were selected from the WT and three transgenic lines, and then four areas per leaf were injected by a syringe with the bacterium *X*. *campestris pv*. *campestris* (*Xcc*) (which causes a black rot disease in crucifer family plants). Five days after inoculation, severe necrosis was found throughout the WT leaves. However, the lesions in leaves from the three transgenic lines were limited to around the injected areas and showed clearly attenuated necrosis (Fig 8B). In whole-plant tests, 4-week-old *Arabidopsis* plants were inoculated with *Xcc* by spraying. In WT plants, significant necrotic lesions appeared in the leaves at day 5 and more severe symptoms developed at day 7; with time, the plants exhibited systemic necrosis, completely collapsed and died at day 10 after inoculation (Fig 8C and 8D). However, transgenic *Arabidopsis* plants expressing *OsPR10a* showed strong tolerance to *Xcc* infection. There were no significant lesion areas found at day 5 and only limited symptoms of necrotic lesions in leaves were found 7 days after inoculation (Fig 8C). *Arabidopsis* plants overexpressing *OsPR10a* showed a survival rate of 35%–70% after 10 days of *Xcc* inoculation, compared with 0% for the wild-type (Fig 8D). Although some leaves of transgenic plants appeared necrotic, many of leaves remained green, exhibited small lesions and continued to grow. To further determine the effect of *OsPR10a* on pathogen defense-related genes expression in *Arabidopsis*. The expression of four defense-related genes [51], *AtCYP*, *AtGST*, *AtERF1*, and *AtWRKY30*, in the *Arabidopsis OsPR10a*-overexpressing lines were analyzed. The levels of *AtCYP*, *AtGST*, *AtERF1*, and *AtWRKY30* mRNAs in *OsPR10a*-overexpressing lines were no significant difference as compared with those of wild type (S6 Fig).

### Overexpression of *OsPR10a* in rice and *Arabidopsis* increased root length under phosphate starvation

**Fig 9:**
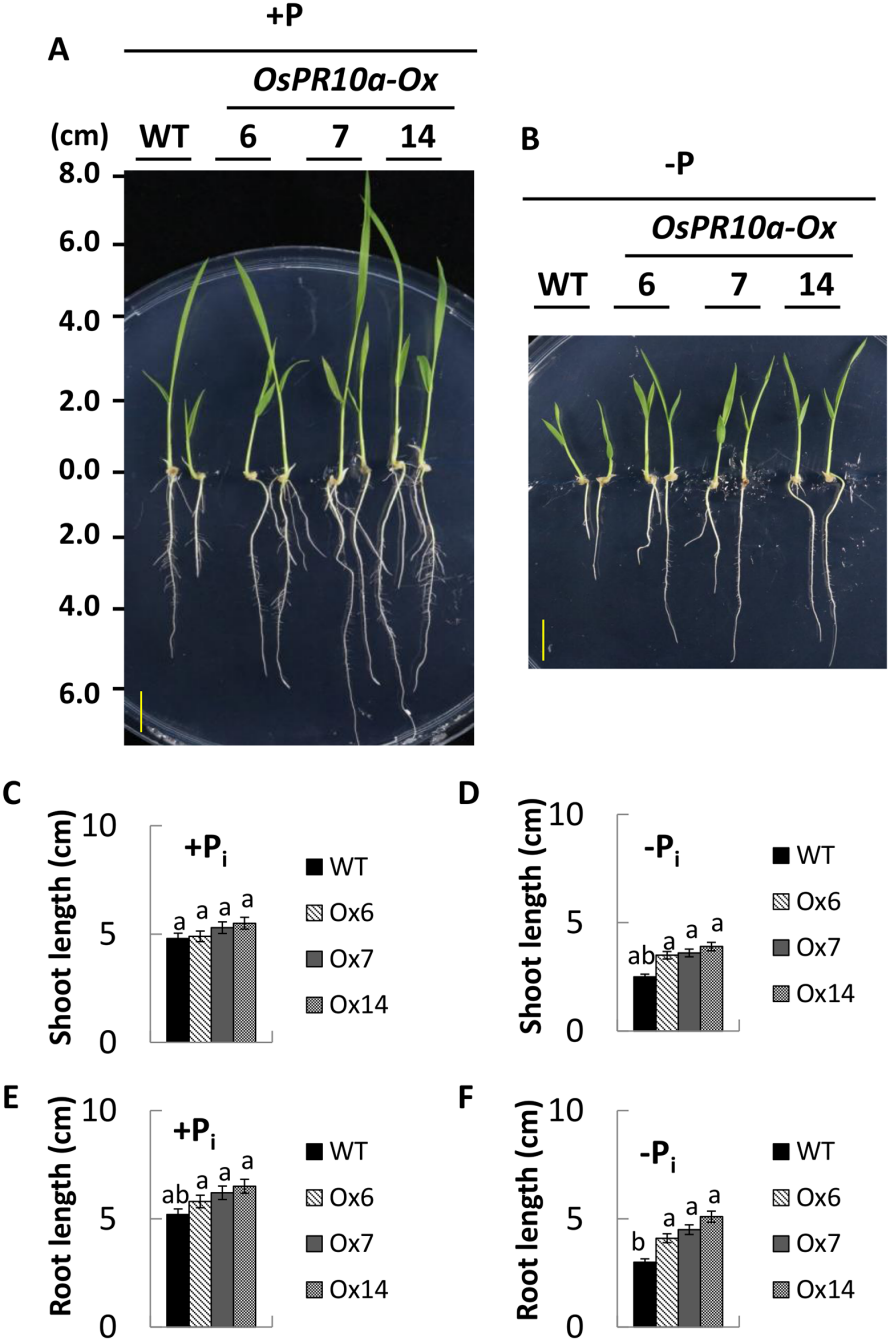
Phenotypes of WT and the *OsPR10a*-overexpressing transgenic seedlings under +P_i_ and –P_i_ conditions. Rice seeds were imbibed in distilled water for 3 days, germinating embryos were isolated and placed onto the vertical plates containing solid half-strength of MS medium supplemented with or without P_i_. (A) and (B) Isolated embryos incubated either in +P_i_ medium (A) or–P_i_ medium (B) for another 6 days were photographed. Bars = 1 cm. (C) and (E) Quantitative analyses of shoot lengths (C) and primary roots (E) of seedlings cultured in +P_i_ medium. (D) and (F) Quantitative analyses of shoot lengths (D) and primary roots (F) of seedlings cultured in -P_i_ medium. Groups that do not share the same letter are significantly different estimated by ANOVA (P <0.05). Data are shown as means ±SD (n = 10).

The *OsPR10a* gene expression and its RNase-related activity were induced by phosphate starvation in suspension-cultured rice cells (Figs 1, 2 and 5). The transgenic *OsPR10a*-overexpressing rice and *Arabidopsis* plants were used to examine the contribution of *OsPR10a* responding to phosphate starvation in plants. Three-day-old of germinating seeds of *OsPR10a*-overexpressing and the wild-type lines were incubated onto a half-strength of Kimura B hydroponic solution [47] supplemented either with 12.4 mg/L KH_2_PO_4_ or not for 12 days. No significant differences were found in root length and plant height between WT and transgenic lines incubated with or without P_i_ (S7 Fig). To eliminate the P_i_ released from endosperm during rice seed germination, embryos were isolated from WT and *OsPR10a* overexpressing transgenic lines of *Ox6*, *Ox7*, and *Ox14* after 3 days of germination (the example shown in S8A Fig). These embryos were cultured onto a vertical plate containing solid half-strength MS medium supplemented either with or without P_i_ for 6 days. No significant differences in plant height and root length between WT and *Ox6* and *Ox7* when grown in +P_i_ medium, although the *Ox14* line exhibited slightly longer root and shoot than those in WT (Fig 9A). However, under -P_i_ condition, lengths of root in *Ox6*, *Ox7*, and *Ox14* were 3.57±0.43 cm, 4.37±0.58 cm, and 4.85±0.49 cm, respectively, that were significantly 19.8%, 46.6% and 62.7% longer than WT plants (2.98±0.25 cm) (Fig 9B and 9F). Meanwhile, the shoot lengths of three *OsPR10a* transgenic lines were also higher than WT (Fig 9B and 9D). These results indicated that constitutive overexpression of *OsPR10a* in rice promoted seedling growth under -P_i_ condition.

**Fig 10:**
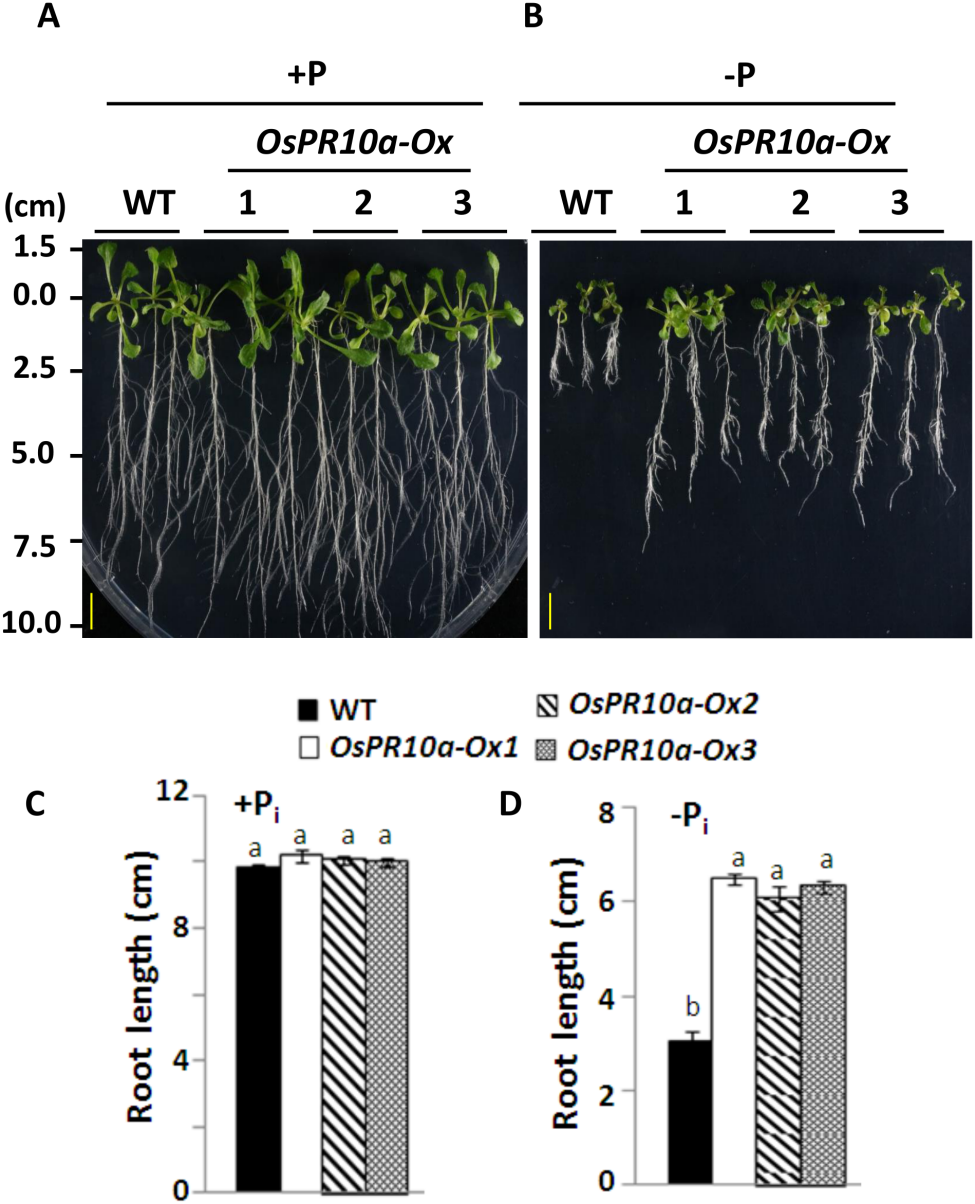
Growth performances of WT and *OsPR10a*-overexpressing in *Arabidopsis* under +P_i_ and –P_i_ conditions. (A) and (B) Phenotype of *Arabidopsis* seedlings cultured either in P_i_-containing or P_i_-free medium. *Arabidopsis* seeds were sterilized and then sowing onto the vertical plates containing solid half-strength of MS medium supplemented with (A) or without (B) P_i_, and then were incubated for 14 days. Bars = 1 cm. (C) and (D) Quantitative analyses of primary root of seedlings cultured in +P_i_ (C) and –P_i_ (D) medium. Groups that do not share the same letter are significantly different estimated by ANOVA (P <0.05). Data are shown as means ±SD (n = 10).

The *OsPR10a* function in *Arabidopsis* response to phosphate starvation was also examined. Seeds of WT and *OsPR10a*-overexpressing lines, *OsPR10a-Ox1*, *-Ox2*, and *-Ox3* were cultured on a vertical plate containing solid half-strength MS medium supplemented either with P_i_ or not for 14 days. As shown in Fig 10A and S9A Fig, a similar phenotype of rosette leaves and root length was observed among WT and transgenic lines when they were grown on +P_i_ medium. However, the primary root lengths of three *OsPR10a* overexpression lines were significantly longer than wild type in -P_i_ condition (Fig 10B and 10D, S9B and S9D Fig). These results also indicated that ectopic expression of *OsPR10a* improved root growth in *Arabidopsis* under P_i_ deficiency.

## Discussion

Nutrient limitation usually has pleiotropic effects on plant development and metabolism; among such types of limitation, that of sugar is thought to be connected to nitrogen and phosphate in terms of coordinating the regulation of cell growth and metabolism [1,52,53]. In this study, the growth of rice cells was retarded under conditions of deficiency of various nutrients, and the order of the extent of the impact was: starvation of sugar (-S) > nitrogen (-N) > phosphate (-P_i_) (S2 Fig), which implies that carbohydrates are the most important nutrient during rice cell growth and macronutrients, for example, nitrogen and phosphate, modulate cell activities. Fig 1A shows that the order of the extent to which nutrient limitation increases RNase activities in rice cells is: -P_i_ > MS > -S = -N. Phosphate starvation induced RNase activities, which allowed phosphate-limited cells to maximize P_i_ acquisition from intra- and extra-cellular components, which are generally derived from RNA molecules, followed by the reprioritization of internal P_i_ use [3–5]. Our results revealed that -P_i_ conditions induced the activity of a 17 kDa RNase in rice cells (Figs 1 and 2), which indicated that this ribonuclease might play a role in hydrolyzing ribonucleic acids in P_i_-limited rice cells. Subsequently, the liberated P_i_ and nucleosides might be transferred into salvage or metabolic pathways.

Amino acid sequencing analysis of the 17 kDa RNase showed that this protein corresponds to OsPR10a (PBZ1), which plays a role in resistance to pathogen infection. Proteins orthologous to OsPR10a are classified into the PR10 family, which is closely related to the Bet v1 superfamily [54]. Bioinformatic analysis showed that the OsPR10a protein has eight negatively charged amino acid residues, and its molecular weight is 16.7 kDa. This is consistent with the Bet v1 superfamily, which consists of small, acidic proteins of 17–18 kDa [12,15]. Structure analysis showed that, in birch Bet v1, there are three critical amino acids, E97, E149 and Y151, and a conserved sequence of a P-loop (G×GG×G××K), which are both essential for the ribonuclease activity [32,55,56]. The rice OsPR10a protein exhibits a conserved sequence, including residues E100, E147 and Y149, and a P-loop-like motif (GNGGPGTIY) located at amino acid positions 44–52 (S10 Fig), so we considered that OsPR10a might have RNase activity. Although OsPR10a (PBZ1) was previously proposed to possess RNase activity [28], further ribonuclease activity analyses of OsPR10a were performed in the present study which showed that RNAs were digested completely within 5–30 min, regardless of whether OsPR10a had been purified from rice cells or was bacterially expressed recombinant protein (Fig 3, S3 Fig). A biochemical assay revealed that the Mg^2+^ was necessary for maintaining the RNase activity of the purified 17 kDa protein, whereas did not require divalent cations Ca^2+^, Mn^2+^ or Co^2+^, and was completely inhibited by Cu^2+^ and Zn^2+^, Hg^2+^ and Ag^+^ (Fig 4B), similar to findings on AmPR10 from *Astragalus mongholicus* [57] and ZmPR10 from *Zea Mays* [31]. Moreover, the RNase activity of bacterial recombinant OsPR10a was inhibited completely by EDTA (S3B Fig), this result also support the notion that Mg^2+^ is required for optimal RNase activity of OsPR10a. The RNase activity of OsPR10a, regardless of whether it was purified from rice cells or *E*. *coli*, was abolished in the presence of the reducing agents β-ME and DTT, so the four cysteine residues that can form disulfide bonds in OsPR10a are proposed to play roles in the maintenance of its RNase activity (Fig 4C, S3B Fig). Similar results have also been reported for other plant RNases whose activities were blocked by reducing agents [58–60].

The expression of some plant ribonucleases in cultured cells is induced upon phosphate starvation [3–5]. Here, we showed that the -P_i_ induced ribonuclease activity of 17 kDa protein is OsPR10a, and its mRNA accumulated at a high level in phosphate-limited rice cells (Figs 1, 2, 5 and 6). To our knowledge, gene expression of PR10 family members has not been reported to be induced by phosphate starvation. Our observations define a new feature of OsPR10a that it is not only functionally correlated with pathogen defense, but also with the recycling of phosphate in response to P_i_ starvation within rice cells. The expression of some genes that encode ribonuclease-like PR10-related proteins is induced during leaf senescence, such as *Ypr10C* in common bean and *OsPR10a*::*GFP* in transgenic rice [27,61], and our study showed that the expression of *OsPR10a*::*GUS* is also upregulated in senescing leaves (Fig 6C), suggesting that OsPR10a and its RNase activity may play a role in leaf senescence to scavenge ribonucleic acids and transport them to growing tissues.

PR10-related genes have been widely studied in various plant species in terms of their gene/protein expression in response to pathogen infection, for example ZmPR10 and ZmPR10.1 in maize leaves, GaPR10 in cotton seedlings, and JIOsPR10 and PBZ1 in rice [12,17,21,27,31]. Infiltration of recombinant purified rice PBZ1 proteins in tobacco leaves or treatment with dexamethasone to induce the expression of PBZ1 in transgenic *Arabidopsis* both induced a hypersensitive response and led to cell death in leaves [28]. However, only a few *in vivo* experiments have been able to show that PR10-related genes could increase resistance to pathogen attack, for example, ectopic expression of maize ZmPR10.1 in *Arabidopsis* against *Pseudomonas syringae* [31], and co-overexpression of pepper PR10 and its interacting protein LRR1 in *Arabidopsis* against *P*. *syringae* and *Hyaloperonospora arabidopsidis* [62]. Here, our results demonstrated that *OsPR10a* gene expression was induced in leaves (Fig 6D) and in cultured cells (Fig 6B) during *Xoo* infection, suggesting that *OsPR10a* may play a role in resistance to *Xoo*. Overexpressing *OsPR10a* in rice and *Arabidopsis* led to high resistance to *Xcc* infection (Figs 7 and 8), although Kim et al. [28] reported that constitutive overexpression of *35S*::*PBZ1* (*35S*::*OsPR10a*) in *Arabidopsis* resulted in a harmful effect on seed germination. However, under the control of the maize ubiquitin promoter to express *OsPR10a* (*Ubi*::*OsPR10a*) (Fig 7A) ectopically in rice and *Arabidopsis*, we found that there were no significant differences in seed setting, seed germination rates, and seedling growth between WT and transgenic plants (S11 Fig). Furthermore, we found that expression of several well-known pathogen resistance relative genes [50,51] were not affected in the *OsPR10a* overexpression rice and *Arabidopsis* neither under pathogen infection nor not (S5 and S6 Figs), suggesting that the OsPR10a is a downstream protein in pathogen resistance mechanism, and contributes plant pathogen defense by its RNase activity. However, the enzyme activity of OsPR10a might be conditional regulated by an unknown process. Under normal growth conditions, the WT and the transgenic *OsPR10a*-overexpressing rice and *Arabidopsis*, all displayed no significant difference during growth and development (S11 Fig). When pathogen infection occurred as shown in Fig 8B, the necrotic regions were limited to bacteria-inoculated areas in transgenic leaves, whereas severe necrosis was observed throughout leaves of the WT, suggesting that a hypersensitive response can be induced by OsPR10a after pathogen attack, resulting in necrotic cell death in order to restrict pathogens to the infected regions. This observation is similar to the dexamethasone-induced expression of rice PBZ1, which causes cell death in transgenic *Arabidopsis* [28]. When whole-plants were spray-inoculated with *Xoo* and *Xcc* in rice and *Arabidopsis*, respectively, the *OsPR10a-Ox* plants also developed much less severe disease symptoms than the WT (Figs 7 and 8). These results indicate that OsPR10a plays a role in plant defense against bacterial pathogens of *Xoo* in rice and *Xcc* in crucifer plants. Several PR10 proteins/genes have been shown to be expressed after wounding [63–65], suggesting roles in defense against mechanical injury caused by herbivory or parasitism. As shown in Fig 6D, GUS expression in rice leaves was induced upon wounding (mechanical stress), implying that OsPR10a might also have a protective role against chewing damage caused by insect pests, in addition to pathogen resistance. Similar results also shown in Fig 5, when rice suspension-cultured cells were transferred to either +P_i_ or—P_i_ media, the levels of *OsPR10a* mRNA were increased transiently in 3 h and 6 h, and then were progressively decreased, suggest that *OsPR10a* might also induced by mechanical stress, such as subculture transfer shock of suspension cells.

In flower plants, the fertilization steps involve mature pollens released from the opening anthers, pollen germination on the surface of stigma and pollen tube growth followed by pass through the transmitting tissue of the style toward the ovary for successful fertilization [66,67]. Therefore, the interactions between the growing pollen tube and the transmitting tract of the style are important in fertilization process. In terms of fertilization on flowering plants, self-incompatibility plays a pivotal role in the evolution and diversification among closely related species [68,69]. In Poaceae, there have been identified a two-locus S and Z system, in which the presence of identical S and Z alleles in pollen and pistil inhibits fertilization [70]. As shown in Fig 6E, strong GUS staining was observed specifically in the anther, style and the style-ovary junction before and right after flowering. By contrast, no GUS staining could be found in the stigma, ovary (Fig 6E), and pollen (data not shown). This spatial and temporal expression of GUS activity might correspond to RNase activity. As described above, the *OsPR10a* expressing *Arabidopsis* plants were still self-fertile and produced normal siliques and seed number per silique, we therefore suggest that the gene might be linked to neither S nor Z locus. If the role of OsPR10a is functionally correlated with fertilization process, the RNase activity might contribute to the anthers opening or to improve pollen tube entering into ovary by the induction of hypersensitive response and programmed cell death. However, we cannot rule out a possible role of OsPR10a during the flowering stages, which may contribute to increase disease resistance and/or to degrade the certain RNAs for supplement of P_i_ to support the growing pollen tube via its RNase activity. To date, there is no report regarding the function of PR10-related gene/protein on seed germination. We found that *OsPR10a*::*GUS* was induced specifically in the germinating embryos and shoots, but not in endosperms and roots (Fig 6F). Whether OsPR10a also plays a role in either defense against pathogen infection and/or P_i_ recycling in germinating rice seeds is required be verified. Our study might also provide clues toward such a novel function of OsPR10a during rice fertilization process and seed germination for future studies.

Plants have an adaptive response through inhibition of primary root growth, and increase formation of lateral roots and production of root hairs to overcome with P_i_ deficiency [71]. Overexpression of a rice WRKY transcription factor, *OsWRKY74*, exhibited higher tolerance to low P_i_ by an increase in biomass of shoot and root, suggested that OsWRKY74 is a positive regulator of P_i_ limitation responses [72]. In the present studies, we found that transgenic *OsPR10a*-overexpressing rice and *Arabidopsis* significantly improved primary roots elongation and slightly increased seedlings growth under P_i_ depletion condition (Figs 9 and 10). These results demonstrated that OsPR10a plays a positive role in rice and *Arabidopsis* seedlings tolerance/adaptation to P_i_ deficiency. The OsPR10a might salvage P_i_ from unwanted RNA via its RNase activity to maintain seedling growth during exogenous P_i_ is limited. However, it is not rule out that the OsPR10a might be through an unknown factor to enhance adaptation of plants to P_i_ starvation.

In this study, we found no significant different phenotype of whole germinated rice seedlings between WT and *OsPR10a*-overexpressing lines under +P_i_ and –P_i_ treatments (S7 Fig). It is possible that rice endosperm supplies sufficient P_i_ during seed germination and early stages of seedling development. Previously, the P_i_ deficiency phenotypes of rice seedlings only can be obtained after 30 days of hydroponic cultivation [72,73]. We isolated germinating rice embryos to examine the effect of P_i_ during rice seedling development. Within a short period of time, *OsPR10a*-overexpressing rice significantly improved root elongation under P_i_ depletion condition (Fig 9, S8 Fig). Thus, the isolated embryos are an excellent material to rule out the effect of P_i_ released from endosperm.

We have identified a ribonuclease-like pathogenesis-related protein/gene, *OsPR10a*, from phosphate-starved rice suspension-cultured cells. An in-gel RNase activity assay demonstrated that OsPR10a exhibits ribonuclease activity. Our experiments demonstrated that the expression of *OsPR10a* in rice was induced upon phosphate starvation, wounding, infection by the pathogen *Xoo*, leaf senescence, anther and style, and germinating embryo and shoot. Ectopic expression of *UbiP*::*OsPR10a* in rice and *Arabidopsis* conferred enhanced resistance to infection by the pathogen *Xoo* and *Xcc*, respectively. Moreover, both *OsPR10a*-overexpression transgenic rice and *Arabidopsis* seedlings significantly increased root lengths on -P_i_ medium. These results reveal multiple roles of *OsPR10a*, which might functionally correlate with the recycling of phosphate in phosphate-starved cells and senescing leaves, and improve resistance to pathogen infection and tolerance to P_i_ deficiency. Similarly, the *Arabidopsis* purple acid phosphatase 5 (PAP5) gene, which has been identified as a regulator of P_i_ uptake, is induced under P_i_ starvation and required for maintaining basal resistance to pathogen infection [74,75]. Our results (S12 Fig) showed that under -P_i_ condition, rice seedlings were more resistant to *Xoo* infection than that grown in +P_i_ hydroponic solution. These results provide a clue that P_i_ acquisition and/or homeostasis may be associated with disease resistance in plants. Our results also show that *OsPR10a* gene expression was induced by both phosphate starvation and pathogen infection, and enhanced resistance to pathogen challenge of *Xoo* in rice and *Xcc* in *Arabidopsis*. These findings therefore provide a good model system via expression of *OsPR10a* to study the mechanisms of the relationship between phosphate starvation and plant-pathogen interactions, and also offering a clue for manipulation of *OsPR10a* in crops disease resistance.

## Supporting Information

S1 Fig OsPR10a genomic DNA sequence. (PDF)

S2 Fig Biomass of rice suspension-cultured cells growth under normal and nutritional starvation of culture media. (PDF)

S3 Fig Purification and characterization of OsPR10a protein expressed in *E*. *coli*. (PDF)

S4 Fig Southern blot analysis of *Ubi*::*OsPR10a* transgenic lines. (PDF)

S5 Fig RT-PCR analysis of pathogen resistant genes in WT and *OsPR10a*-overexpressing lines. (PDF)

S6 Fig RT-PCR analysis of pathogen resistant genes in WT (*Col-0*) and *OsPR10a*-overexpressing lines of *Arabidopsis*. (PDF)

S7 Fig Hydroponic culture of *OsPR10a*-overexpressing rice seedlings under +P_i_ and –P_i_ conditions. (PDF)

S8 Fig Phenotypes of WT and *OsPR10a*-overexpressing transgenic lines under +P_i_ and –P_i_ conditions. (PDF)

S9 Fig Phenotype of WT and *Arabidopsis OsPR10a*-overexpressing grown in +P_i_ and –P_i_ conditions. (PDF)

S10 Fig Amino acids sequence of OsPR10a. (PDF)

S11 Fig Comparison of the phenotypes of the WT and the transgenic lines under normal growth conditions. (PDF)

S12 Fig Enhanced disease resistance against *Xoo* challenge under -P_i_ conditions in WT rice seedlings. (PDF)

S1 Table Primers and their sequences used in this study. (PDF)
