# Supplementary material for: Multiple Patterns of Regulation and Overexpression of a Ribonuclease-Like Pathogenesis-Related Protein Gene, OsPR10a, Conferring Disease Resistance in Rice and Arabidopsis
Source: PLoS One. 2016 Jun 3;11(6):e0156414. doi: 10.1371/journal.pone.0156414 (PMC4892481; doi:10.1371/journal.pone.0156414)
Supplement: S1 Fig — (PDF) [file pone.0156414.s001.pdf]

```
-1350  CCGCCGCCGC CGTGGCGCTC AGGGTGCATG GTTGCGACCA TTTTGGCGCC
-1300  TACTTCTCGC GGAGGCCGGC GAGGTGCACG CTCGATGGCG CCGACGTGGG
-1250  GTTCACCTAC GACGGCGACA CGAGGACATG CTCACAGAGG GACCCGCGTC
-1200  GGATTAATTT GAGCCAAGGG AAAACTGGTC TTTAATGATT ATTTCTCTCT
-1150  CCTATTCCTC CAAAAATAAT AAAATAATCT CACCGGTGTC CAGCAGCTAA
-1100  TTAACAGTTT TTTGTGATGC CCTACAGCAA ATACACGTTC TTACGACATC
-1050  CCCTAGTTAA TTACACGTTT TTCGGACGTC CTGTAGCAAA TTTTGCCGTA
-1000  TAACAATTAC ATTACTATCC TCATCTTTCC AAACCAAGGG TAGGAAGTAG
 -950  AGAACTTCTC TAAAAGTGTT TTGGAATGTA AGAACATATT TTGACACAAG
 -900  TTTTGAATGC TGGAATGATA AGCAATTTGA AACGGAGAGA TTTATCAAAG
 -850  TTAGGACGTA CGTGCTCTGG TACTAGCCGT ACGATGACGC CCAATAATTC
 -800  AACCGAAGAA CAACCACACC TATCGATCCG AGGTGGCAAG GTGGAAATTT
 -750  TGCGTTAAAG CTCAATTTGT CCCTGGTGAC CGTGACATCA GATTGAGTAT
 -700  CACTGAGTCT ACCAATTGAA GGTTGTATAT ATCCGAGGTG GCACAGTGAA
 -650  AATTGATACG CTATGAAACC CAACAAGATT GAAAGAAATT CATAATTGAA
 -600  TTAATACCTA CCGATAAAGG GTATTTGTTT AGACCCATCT CAGAGCATGA
 -550  CATGTAGTCG TACCTATCAT CTAAAAGCAT TTAAATTAGG GTCTGTTCGA
 -500  TTTAGATTAT TAAACAAATT ATTATCGTTG ATTACCTACC AATTGATTAT
 -450  GGAAATAAAT TAAATACTTT AAAATTAAAC TTAATAAATA GTTTAAAACA
 -400  AGTGATCAAA GCAGTAGAAT AAAGTTTGTT GAGAGATTTT TTGAAACATA
 -350  GAACAAATAA TCAGTTCCAA TAATCCGGCG AATAATCTGA GAATCAGTGT
 -300  TCTAACTGTA AACAAAGACC ATGATCTCAT ATATGATTAT TCTCCCAACC
 -250  GTCCTATATA TGCCCAGGTC TCAAATGTCA GCTCTTCTAG ATGGAACCAA
 -200  AGAAAAAAAC CCTTAATTTC CACAGGTCAA GCCACATGTG ATCCCCAATA
 -150  TTCCTACTTC CAGAACCCTA GAATTCCACA CAAAGTTCAG CATATGCAAC
 -100  CAATGGAGCT GAGTTCCCAA CTGCAACATT TATTCTGGAT GATGTCTTCT
  -50  TCTCCTCTTG CCACCCTATA AATAGCCCAT GCTACTGCTC ACCTTTGAAG
    1  CACAAGCACA AGCACAAGCA GCTCTAGCTA GCTACAGGCA TCAGTGGTCA
   51  GTAGAGTGAT CAGTTGCAAC TAGCTAGCTA GTTAGATTAT ATCTTCAGTG
  101  ATGGCTCCGG CCTGCGTCTC CGACGAGCAC GCCGTCGCGG TGTCGGCGGA
  151  GCGGCTGTGG AAGGCGTTCA TGGACGCGTC CACTTTGCCC AAGGCCTGCG
  201  CCGGCTTGGT CGACGACATT GCGGTCGAGG GGAACGGTGG TCCGGGCACC
  251  ATCTACACCA TGAAGCTTAA CCCTGGTAGG TCCAGAAAGA TCTAAGTACT
  301  TGTATCTACT GATTGTACTT ATTATCTCGG CCGATTTTTT TTCTTAAATT
  351  TTTGTGAATT TGGTCAAAAT TTAGTCAAAT TCAGTTAAAT TATTTTCAAA
  401  TTTCTGAAAA AAAAATCAGT CCAAAAAGTG CCGAAAATCC CGAAATTTTG
  451  GTTCTACCAA AATGGCTTTC GGCGAAATCG AAAGTGAAAA TCCTGTCAAC
  501  AACAAAAAGA ATTTGATTAA GACAGTTAAA ATTACTATAG TGCAGTATAA
  551  AATTGATTGG GTATATAATA ACAACAAATG TTAAAATTAT ATGCAGCCGC
  601  GGGTGTGGGA AGCACATACA AGACCCGGGT GGCGGTGTGC GACGCCGCAA
  651  GTCATGTCCT AAAGTCGGAT GTGCTCGAGG CAGAAAGCAA GGTGGGGAAG
  701  CTCAAGTCAC ACTCGACGGA GACGAAGCTT GAGGCCACCG GCGATGGCTC
  751  CTGTGTGGCC AAGCTCAAGG TGGAGTACGA GCTCGAGGAC GGCAGCTCAC
  801  TGTCGCCGGA GAAGGAGAAG GACATCGTGG ATGGCTACTA TGGCATGCTC
  851  AAGATGATCG AGGACTACCT CGTCGCTCAC CCTGCCGAAT ACGCCTAAGA
  901  TGAAGAGGAA TACTGCCTCT ATCCAGTATA TCCCACCTAG AGTGAGTGAT
```

S1 Fig. OsPR10a genomic DNA sequence. Based on accession no. D82066, the OsPR10a genomic DNA sequence are indicated as: red letters for TATA box; pink for coding region; blue for intron; and green for stop codon.
