# Supplementary material for: Multiple Patterns of Regulation and Overexpression of a Ribonuclease-Like Pathogenesis-Related Protein Gene, OsPR10a, Conferring Disease Resistance in Rice and Arabidopsis
Source: PLoS One. 2016 Jun 3;11(6):e0156414. doi: 10.1371/journal.pone.0156414 (PMC4892481; doi:10.1371/journal.pone.0156414)
Supplement: S2 Fig — (PDF) [file pone.0156414.s002.pdf]

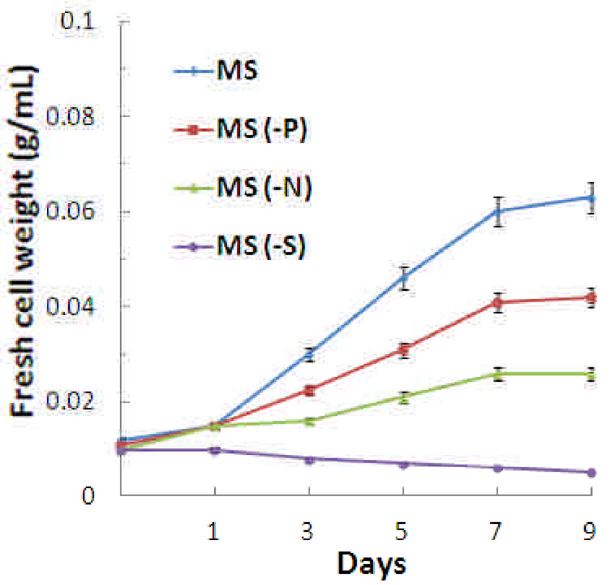

S2 Fig. Biomass of rice suspension-cultured cells grown under normal or nutritional starvation of culture media. Cells were grown in MS completed medium (MS), or MS deficient in either phosphate (-P), nitrogen (-N), or sucrose (-S). Cells were collected at interval time periods of 1, 3, 5, 7, and 9 days, respectively, and fresh weight of cells was measured. Error bars indicate standard errors for the measurements for at least three individual experiments.
