# Supplementary material for: Multiple Patterns of Regulation and Overexpression of a Ribonuclease-Like Pathogenesis-Related Protein Gene, OsPR10a, Conferring Disease Resistance in Rice and Arabidopsis
Source: PLoS One. 2016 Jun 3;11(6):e0156414. doi: 10.1371/journal.pone.0156414 (PMC4892481; doi:10.1371/journal.pone.0156414)
Supplement: S3 Fig — (PDF) [file pone.0156414.s003.pdf]

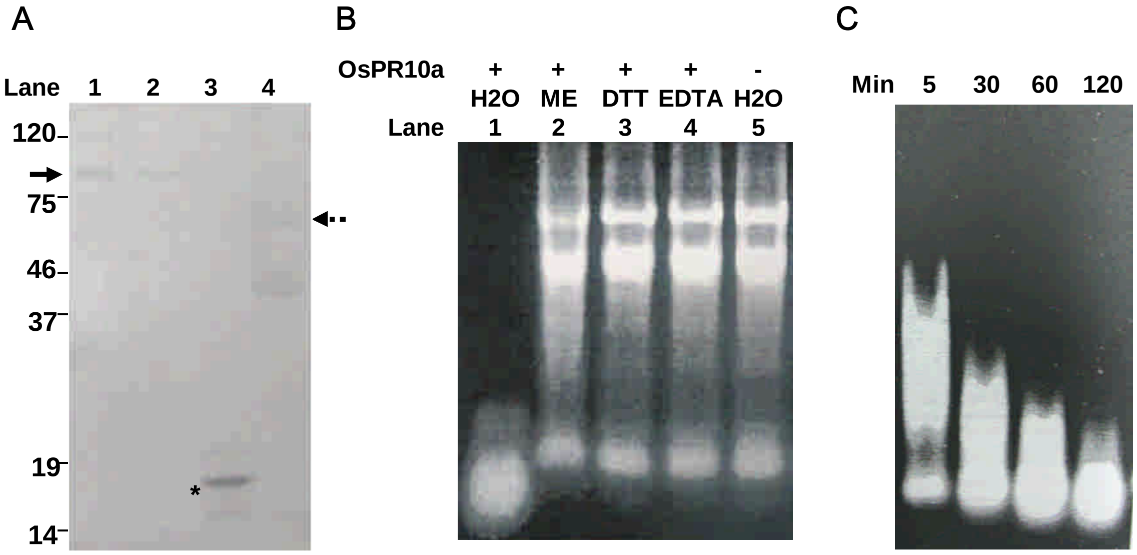

S3 Fig. Purification and characterization of OsPR10a protein expressed in *E. coli*. (A) SDS-PAGE analysis of NusA-tagged recombinant OsPR10a proteins followed by silver staining. The recombinant protein which has a predicted molecular weight of approximately 83 kDa was indicated by a solid arrow at lane 1 (0.2 μg) and lane 2 (0.1 μg). The asterisk (lane 3) indicates OsPR10a protein (17 kDa) that was separated from the NusA-tag using enterokinase. The NusA-tag (66 kDa) is labeled with a dashed arrow in lane 4. (B) RNase activity assay was performed in reaction buffer (25.0 mM sodium acetate, pH 5.0; 2.0 mM $MgCl_2$) containing 1.0 μg of OsPR10a protein and 10.0 μg of rice total RNA as a substrate, supplemented with H_2O, 5% β-ME, 10 mM DTT or 10 mM EDTA, and incubated at 37°C for 2 h followed by agarose gel electrophoresis. (C) The reaction mixture containing 1.0 μg of OsPR10a protein and 10.0 μg of rice total RNA was incubated at 37°C for 5–120 min, and RNase activity was confirmed by agarose gel electrophoresis.
