# Supplementary material for: Multiple Patterns of Regulation and Overexpression of a Ribonuclease-Like Pathogenesis-Related Protein Gene, OsPR10a, Conferring Disease Resistance in Rice and Arabidopsis
Source: PLoS One. 2016 Jun 3;11(6):e0156414. doi: 10.1371/journal.pone.0156414 (PMC4892481; doi:10.1371/journal.pone.0156414)
Supplement: S4 Fig — (PDF) [file pone.0156414.s004.pdf]

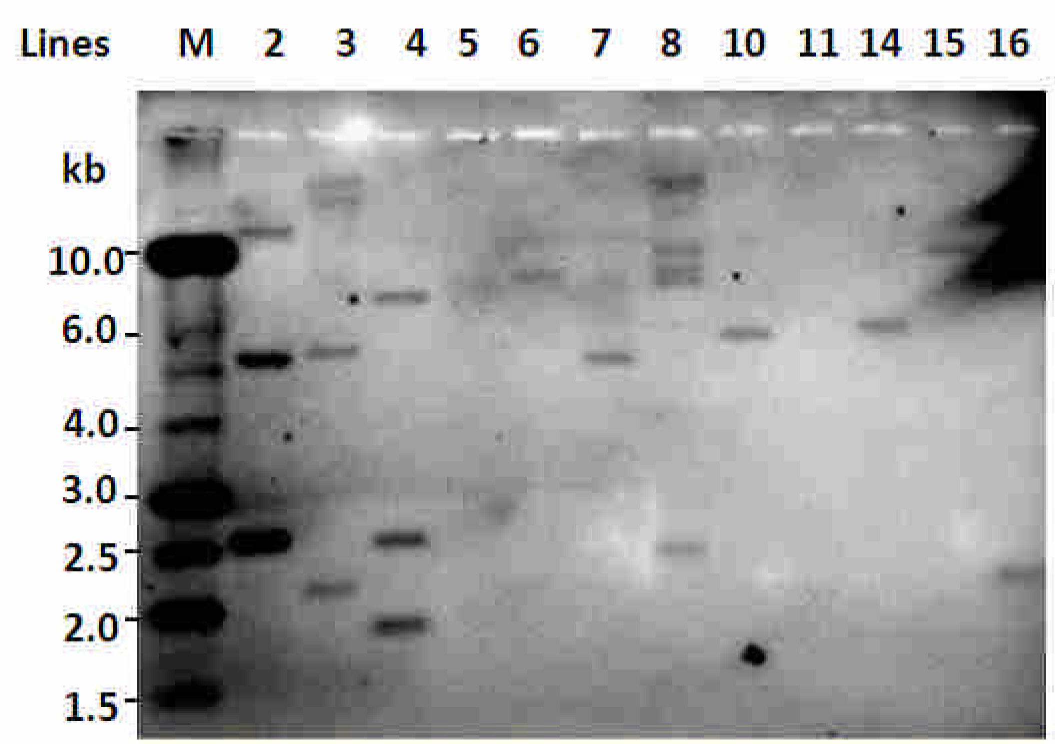

S4 Fig. Southern blot analysis of *Ubi::OsPR10a* transgenic lines. Rice genomic DNA was digested with *Pst*I and subjected to Southern-blot analysis by using the coding region of an antibiotic resistant gene, *Hph* (*hygromycin phosphotransferase*), as a probe.
