# Supplementary material for: Multiple Patterns of Regulation and Overexpression of a Ribonuclease-Like Pathogenesis-Related Protein Gene, OsPR10a, Conferring Disease Resistance in Rice and Arabidopsis
Source: PLoS One. 2016 Jun 3;11(6):e0156414. doi: 10.1371/journal.pone.0156414 (PMC4892481; doi:10.1371/journal.pone.0156414)
Supplement: S5 Fig — (PDF) [file pone.0156414.s005.pdf]

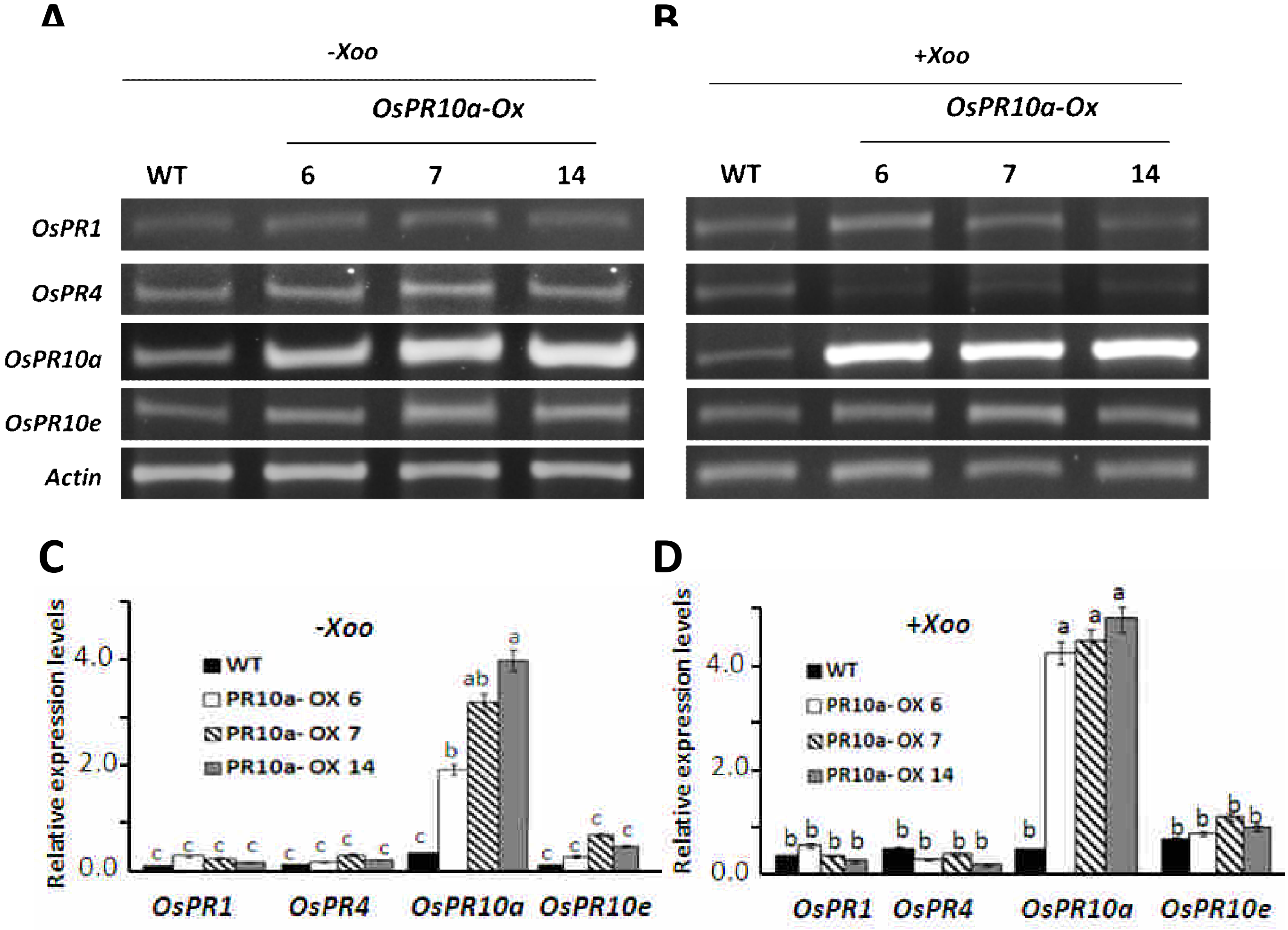

S5 Fig. RT-PCR analysis of pathogen resistant genes in WT and *OsPR10a*-overexpressing lines. The 3-leaf stage of rice seedlings from WT and transgenic lines were inoculated without (A) or with (B) *Xoo* (1.0×10^{8} CFU/mL). After 5 days of infection, the third leaf from each infected plants were isolated followed by RNA purification and proceed the RT-PCR experiments. Expression of *Actin* is shown as a loading control for all samples. (C) and (D) represent the RT-PCR quantification of each gene expression level relative to *Actin.* Groups that do not share the same letter are significantly different estimated by ANOVA (P <0.01). The mean ±SE for three technical repeats. Accession number of the genes are listed in supplemental table 1.
