# Supplementary material for: Multiple Patterns of Regulation and Overexpression of a Ribonuclease-Like Pathogenesis-Related Protein Gene, OsPR10a, Conferring Disease Resistance in Rice and Arabidopsis
Source: PLoS One. 2016 Jun 3;11(6):e0156414. doi: 10.1371/journal.pone.0156414 (PMC4892481; doi:10.1371/journal.pone.0156414)
Supplement: S6 Fig — (PDF) [file pone.0156414.s006.pdf]

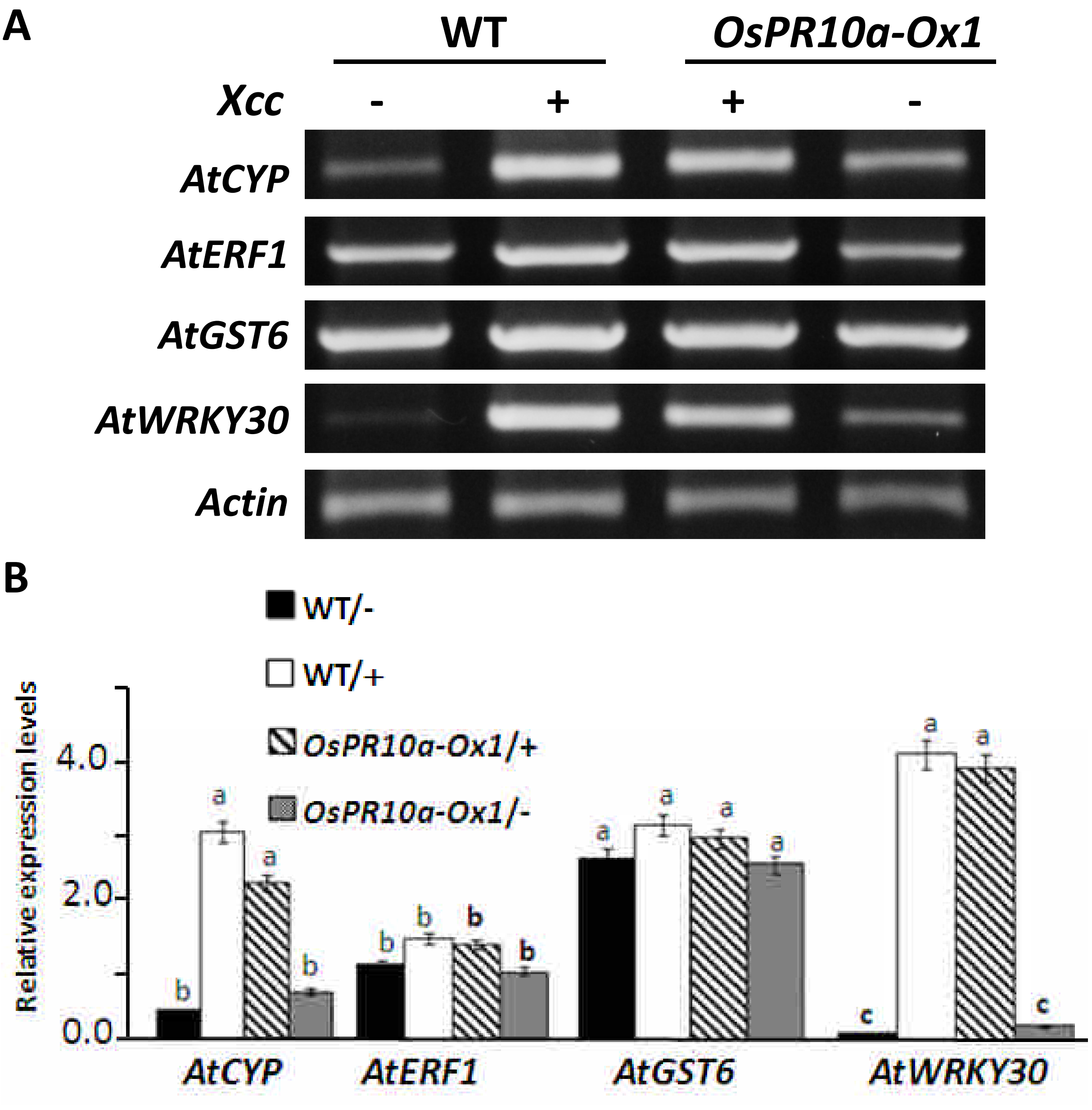

S6 Fig. RT-PCR analysis of pathogen resistant genes in WT (*Col-0*) and *OsPR10a*-overexpressing lines of *Arabidopsis*. The 15-d-old of *Arabidopsis* plants from WT and transgenic lines were inoculated with or without *Xcc* (1.0×10^{8} CFU/mL) by spraying method. After 3 days of infection, the rosette leaves from each infected plants were isolated, followed by RNA purification and proceed the RT-PCR experiments. (A) RT-PCR analysis of pathogen resistant genes were performed. (B) Quantification of each selected gene expression level was calibrated with *Actin.* Groups that do not share the same letter are significantly different estimated by ANOVA ($P<0.01$). The mean ±SE represents for three technical repeats. Accession number of the genes are listed in supplemental table 1.
