# Supplementary material for: Multiple Patterns of Regulation and Overexpression of a Ribonuclease-Like Pathogenesis-Related Protein Gene, OsPR10a, Conferring Disease Resistance in Rice and Arabidopsis
Source: PLoS One. 2016 Jun 3;11(6):e0156414. doi: 10.1371/journal.pone.0156414 (PMC4892481; doi:10.1371/journal.pone.0156414)
Supplement: S7 Fig — (PDF) [file pone.0156414.s007.pdf]

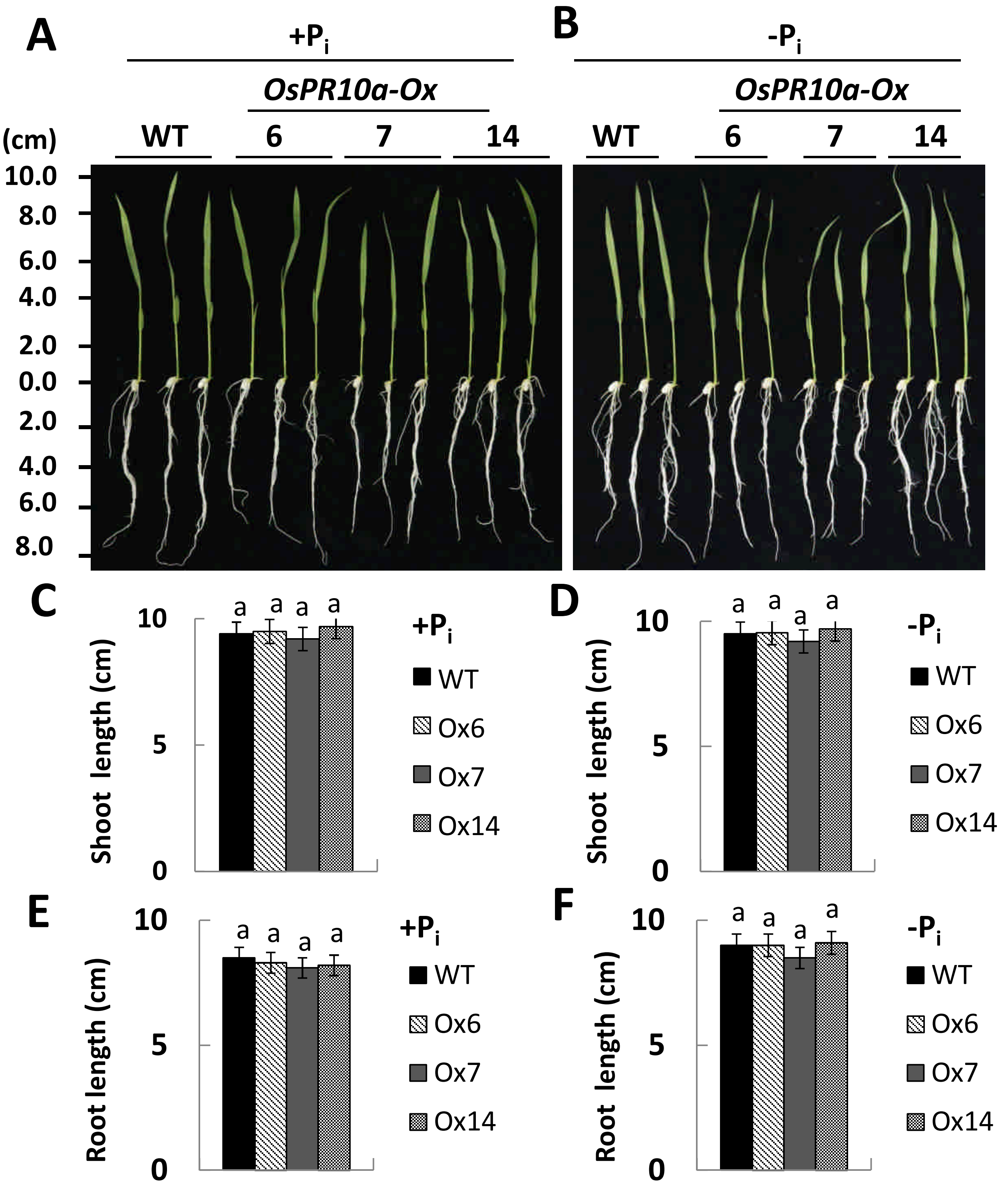

S7 Fig. Hydroponic culture of *OsPR10a*-overexpressing rice seedlings under +P_i and –P_i conditions. Rice seeds were germinated in water for 3 days. Germinating seeds with 2 mm length of primary roots were selected and then placed onto a net floating on a half-strength of Kimura B solution either with (A) or without (B) P_i, respectively. This solution was refreshed every 3 days. After grown for 12 days, rice seedlings were harvested for measurement of shoot and root lengths, and photographed. (C) and (E) Quantitative analyses of shoot length (C) and primary root length (E) of seedlings cultured in +Pi medium. (D) and (F) Quantitative analyses of shoot length (D) and primary root length (F) of seedlings cultured in -Pi medium. Groups that share the same letter are not significantly different estimated by ANOVA ($P < 0.05$). Data are shown as means ±SD (n=20).
