# Supplementary material for: Multiple Patterns of Regulation and Overexpression of a Ribonuclease-Like Pathogenesis-Related Protein Gene, OsPR10a, Conferring Disease Resistance in Rice and Arabidopsis
Source: PLoS One. 2016 Jun 3;11(6):e0156414. doi: 10.1371/journal.pone.0156414 (PMC4892481; doi:10.1371/journal.pone.0156414)
Supplement: S8 Fig — (PDF) [file pone.0156414.s008.pdf]

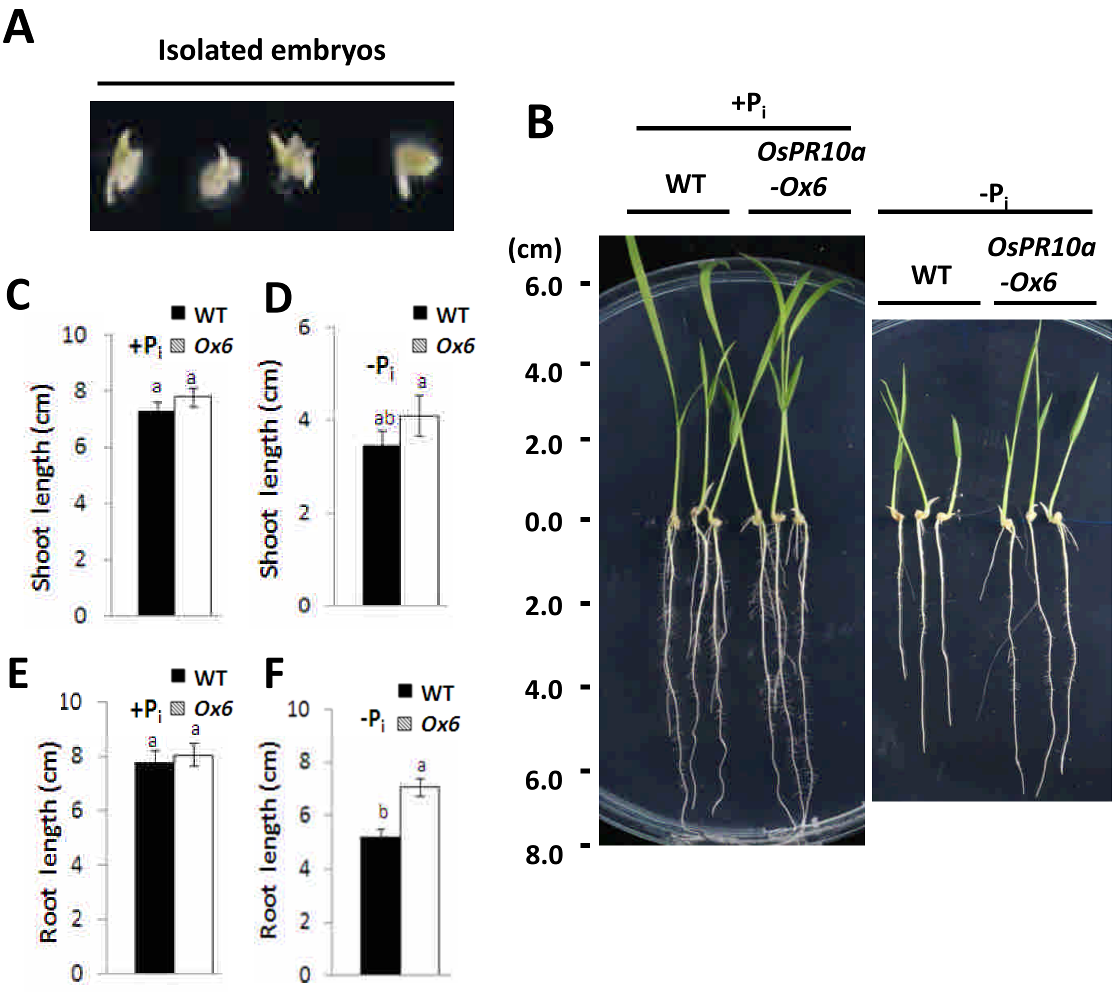

S8 Fig. Phenotypes of WT and *OsPR10a*-overexpressing transgenic lines under +Pi and –Pi conditions. (A) and (B) Rice seeds were imbibed in distilled water for 3 days, germinating embryos were isolated and placed onto the vertical plates containing solid half-strength of MS medium supplemented with (A) or without (B) P_i, and then were incubated for another 8 days. (C) and (E) Quantitative analyses of shoot length (C) and primary root length (E) of seedlings cultured in +Pi medium. (D) and (F) Quantitative analyses of shoot length (D) and primary root length (F) of seedlings cultured in -Pi medium. Groups that share the same letter are not significantly different estimated by ANOVA ($P < 0.05$). Data are shown as means ±SD (n=20).
