# Supplementary material for: Multiple Patterns of Regulation and Overexpression of a Ribonuclease-Like Pathogenesis-Related Protein Gene, OsPR10a, Conferring Disease Resistance in Rice and Arabidopsis
Source: PLoS One. 2016 Jun 3;11(6):e0156414. doi: 10.1371/journal.pone.0156414 (PMC4892481; doi:10.1371/journal.pone.0156414)
Supplement: S9 Fig — (PDF) [file pone.0156414.s009.pdf]

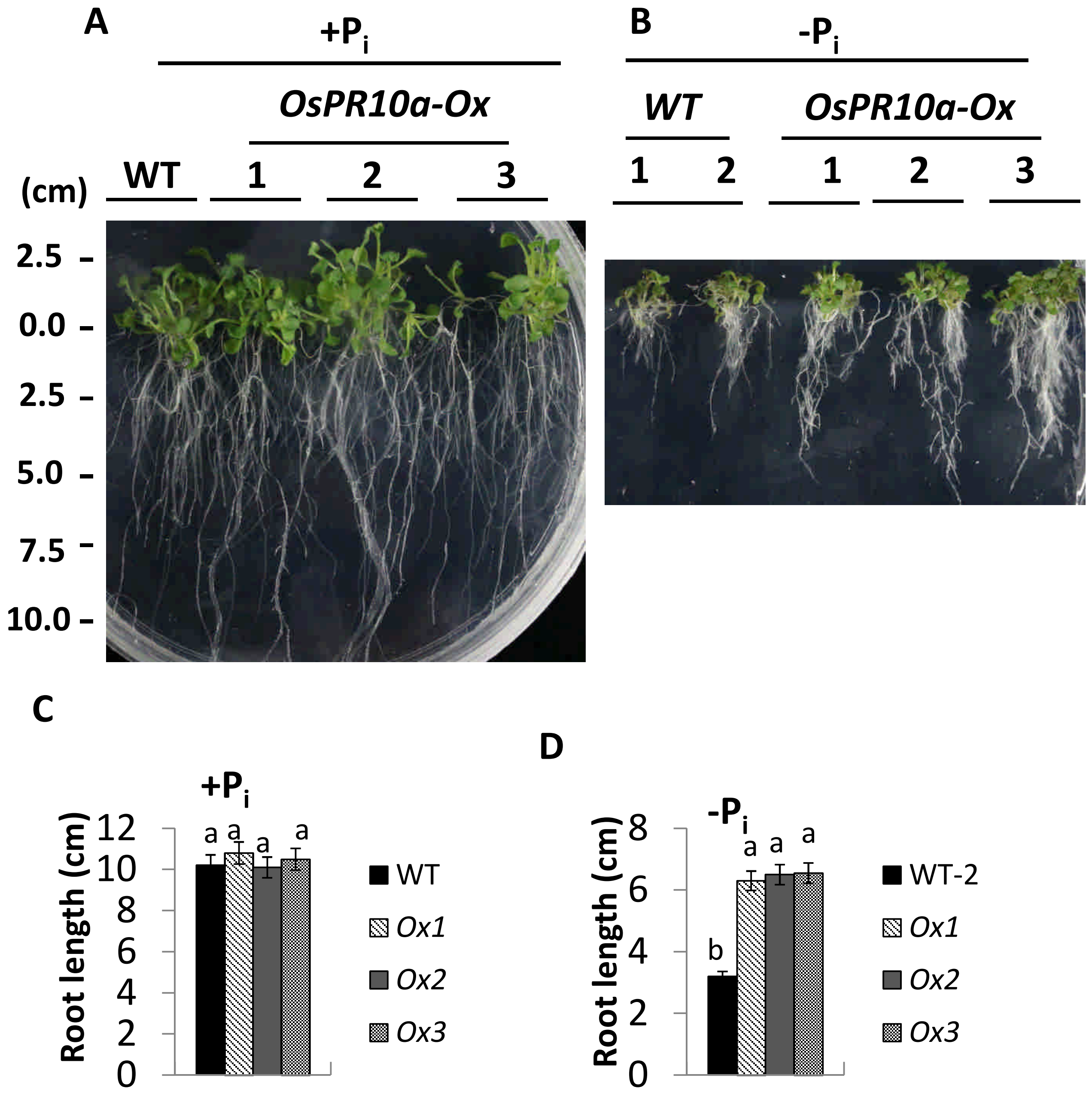

S9 Fig. Phenotype of WT and *Arabidopsis OsPR10a*-overexpressing grown in +Pi and –Pi conditions. (A) and (B) *Arabidopsis* seeds were sterilized and then sown onto the vertical plates containing solid half-strength of MS medium supplemented with (A) or without (B) P_i, and then were incubated for 16 days. (C) and (D) Quantitative analyses of primary root lengths for seedlings cultured in +Pi (C) and –Pi (D) medium. Groups that do not share the same letter are significantly different estimated by ANOVA ($P < 0.05$). Data are shown as means ±SD (n=20).
