# Supplementary material for: Multiple Patterns of Regulation and Overexpression of a Ribonuclease-Like Pathogenesis-Related Protein Gene, OsPR10a, Conferring Disease Resistance in Rice and Arabidopsis
Source: PLoS One. 2016 Jun 3;11(6):e0156414. doi: 10.1371/journal.pone.0156414 (PMC4892481; doi:10.1371/journal.pone.0156414)
Supplement: S10 Fig — (PDF) [file pone.0156414.s010.pdf]

```
  1   MAPACVSDEH AVAVSAERLW KAFMDASTLP KACAGLVDDI AVEGNGGPGT
 51   IYTMKLNPAA GVGSTYKTRV AVCDAASHVL KSDVLEAESK VGKLKSHSTE
101   TKLEATGDGS CVAKLKVEYE LEDGSSLSPE KEKDIVDGYY GMLKMIEDYL
151   VAHPAEYA
```

S10 Fig. Amino acid sequence of OsPR10a. Red letters indicate a P-loop-like motif; and pink letters indicate amino acid residues predicted as Bet v1-like consensus residues. Both conserved sequences are essential for the ribonuclease activity.
