# Supplementary material for: Multiple Patterns of Regulation and Overexpression of a Ribonuclease-Like Pathogenesis-Related Protein Gene, OsPR10a, Conferring Disease Resistance in Rice and Arabidopsis
Source: PLoS One. 2016 Jun 3;11(6):e0156414. doi: 10.1371/journal.pone.0156414 (PMC4892481; doi:10.1371/journal.pone.0156414)
Supplement: S11 Fig — (PDF) [file pone.0156414.s011.pdf]

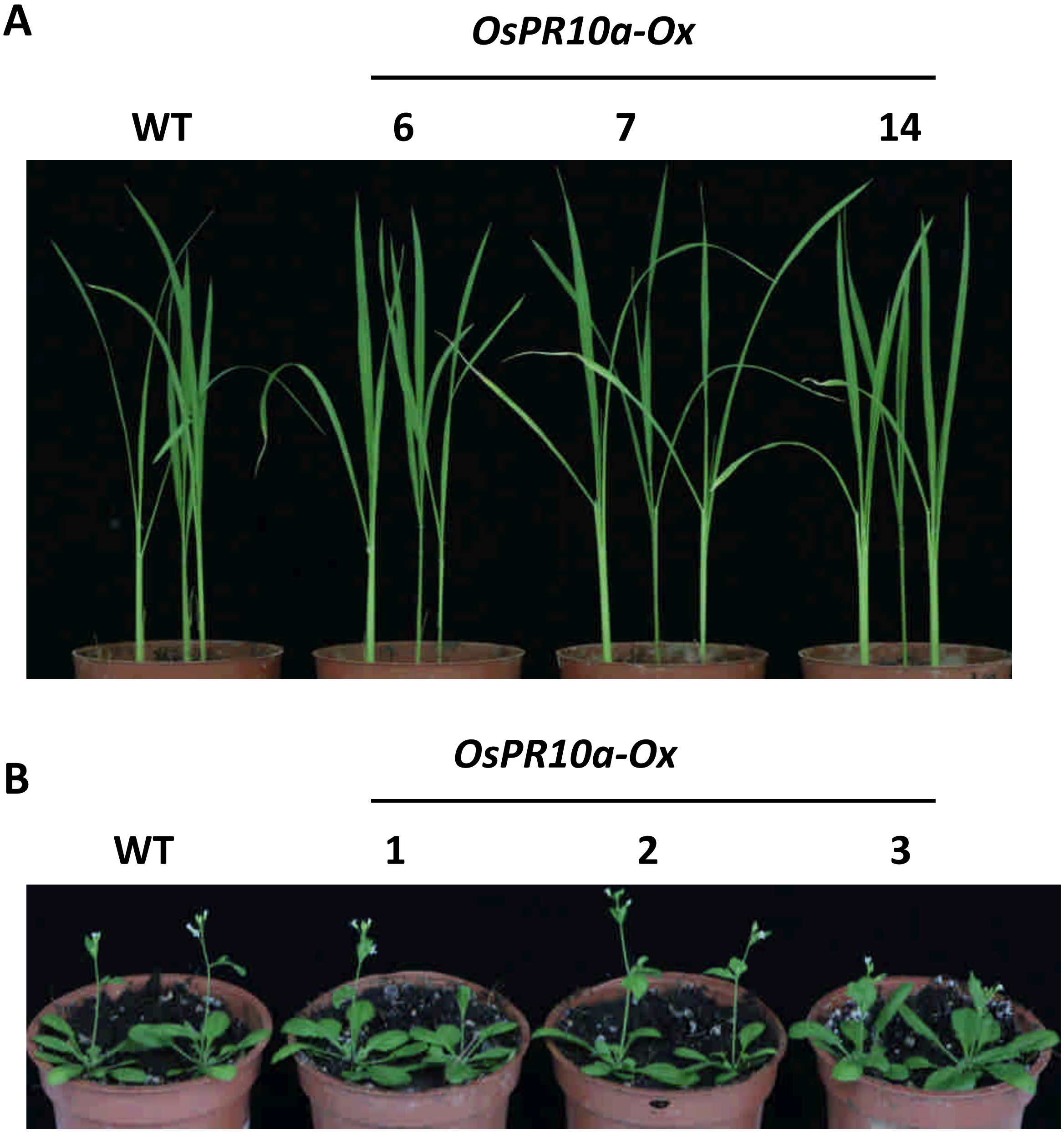

S11 Fig. Comparison of the phenotypes of the WT and the transgenic lines under normal growth conditions. (A) Phenotype comparison between WT and rice *OsPR10a*-overexpressing lines (ox-6,ox-7, and ox-14) cultured in soil under 28℃ for 21 days. (B) Phenotype comparison between WT and *Arabidopsis OsPR10a*-overexpressing lines (ox-1,ox-2, and ox-3) cultured in soil under 22℃ for 21 days.
