# Supplementary material for: Multiple Patterns of Regulation and Overexpression of a Ribonuclease-Like Pathogenesis-Related Protein Gene, OsPR10a, Conferring Disease Resistance in Rice and Arabidopsis
Source: PLoS One. 2016 Jun 3;11(6):e0156414. doi: 10.1371/journal.pone.0156414 (PMC4892481; doi:10.1371/journal.pone.0156414)
Supplement: S12 Fig — (PDF) [file pone.0156414.s012.pdf]

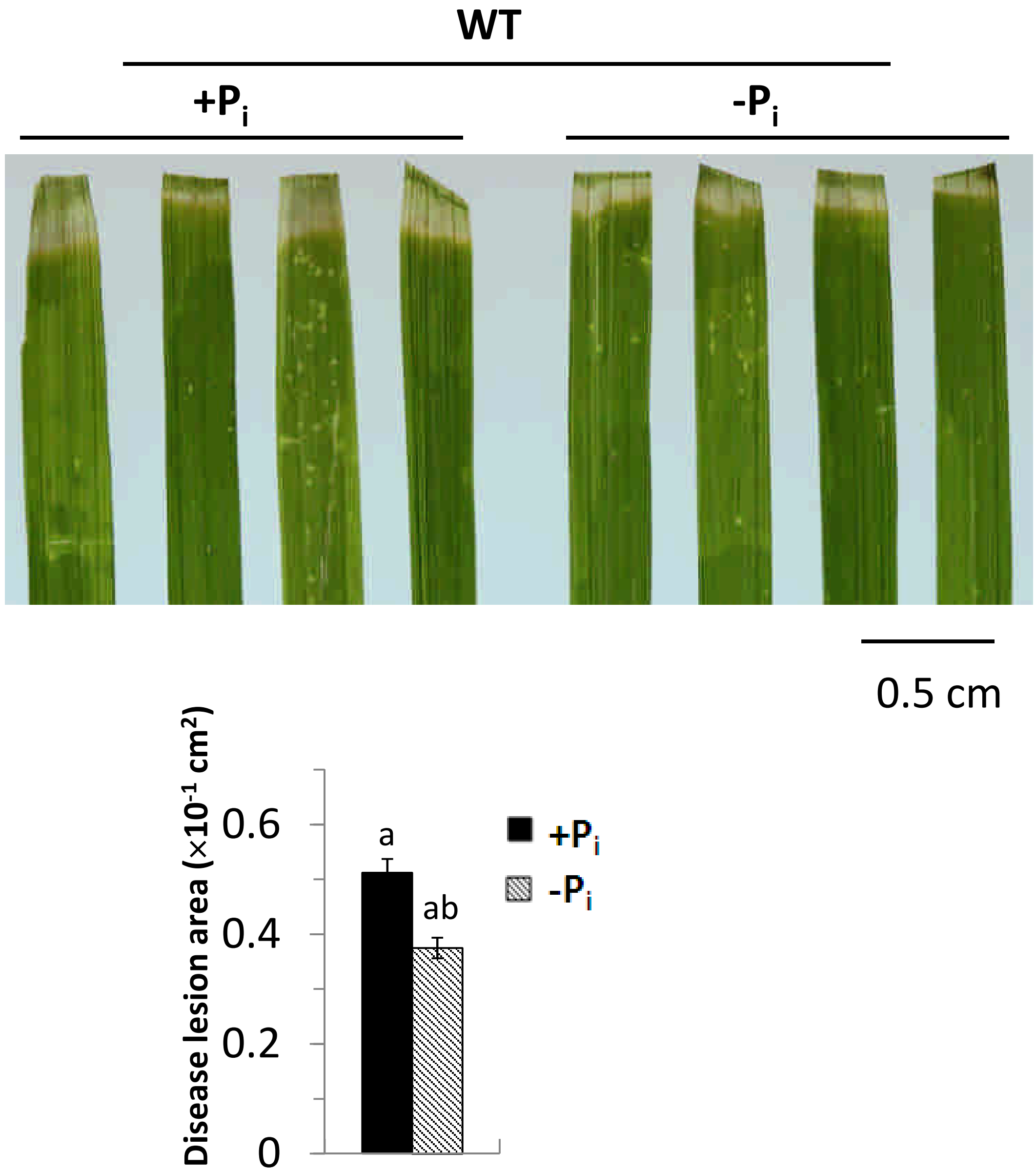

S12 Fig. Enhanced disease resistance against *Xoo* challenge under -P_i conditions in WT rice seedlings. Endosperm of ten-days-old seedlings were removed, and then transferred into half-strength of Kimura B hydroponic solution supplemented either with (+Pi) or without P_i (-Pi), and then were cultured for another 15 days. The 0.5 cm leaf tip was cut from third leaf and then infected with *Xoo* (1.0×10^8 CFU/mL) by a spraying method. After 6 d of inoculation, the infected leaves were isolated and photographed. Groups that do not share the same letter are significantly different estimated by ANOVA ($P < 0.05$). Data are shown as means ±SD (n=10).
