# Supplementary material for: Multiple Patterns of Regulation and Overexpression of a Ribonuclease-Like Pathogenesis-Related Protein Gene, OsPR10a, Conferring Disease Resistance in Rice and Arabidopsis
Source: PLoS One. 2016 Jun 3;11(6):e0156414. doi: 10.1371/journal.pone.0156414 (PMC4892481; doi:10.1371/journal.pone.0156414)
Supplement: S1 Table — (PDF) [file pone.0156414.s013.pdf]

S1 Table Primers and their sequences used in this study.

Name	Sequence
OsPR10aP-5P	5′-ATTCTGCAGCGTGGCGCTCAGGGTGCATG-3′
OsPR10aP-3B	5′-ACTGGATCCACTGAAGATATAATCTAACT-3′
OsPR10a-5P	5′-AATCTGCAGATGGCTCCGGCCTGCGTCTC-3′
OsPR10a-3P	5′-AATCTGCAGTTAGGCGTATTCGGCAGGGT-3′
OsPR10a-RT5	5′-ATGGCTCCGGCCTGCGTCTC-3′
OsPR10a-RT3	5′-GGCGTATTCGGCAGGGTGAG-3′
OsPR1-RT5	5′-ATGGCAACCTCCAGCTTGCT-3′
OsPR1-RT3	5′-CTAGTAGGGAGATTCGCCGT-3′
OsPR4-RT5	5′-ATGGCGGGGATCACCGGATC-3′
OsPR4-RT3	5′-TCAACAATTGACGAACTTGT-3′
OsPR10e-RT5	5′-ATGAAGAGCCTGCAAGGTGAG-3′
OsPR10e-RT3	5′-CTAGTTAGTGGCTTCGCAAGT-3′
AtCYP-RT5	5′-GATCTTATGGACAAAGTGGT-3′
AtCYP-RT3	5′-GCAAGATTTATCCCTGGACA-3′
AtGST6-RT5	5′-ATGGGAGCAATTCAAGCTCG-3′
AtGST6-RT3	5′-TAGCCTCGTAGACATCCAAG-3′
AtERF1-RT5	5′-GAACGACTCAGAGGAAATGT-3′
AtERF1-RT3	5′-ACCACTTCAAACTTAAGGTC-3′
AtWRKY30-RT5	5′-ATGGAGAAGAACCATAGTAG-3′
AtWRKY30-RT3	5′-CATGTCAAGATTTCCGTGTT-3′
AtACT-RT5	5′-ATGGCTGATGGTGAAGACATTC-3′
AtACT-RT3	5′-AGTAGAATAGGATGTTCCTCAG-3′

Gene accession number: *OsPR1* (AF306651); *OsPR4* (AY050642); *OsPR10a* (D82066);
OsPR10e (NM_001068198) ;
AtCYP (AK118875); *AtGST6* (AF288176); *AtERF1* (AF076277)
AtWRKY30 (BT005295); *AtACT* (NM_179953)

Restriction enzyme recognition sites were underlined.
